# MF59^®^-Adjuvanted H5N1 Vaccine Induces Immunologic Memory and Heterotypic Antibody Responses in Non-Elderly and Elderly Adults

**DOI:** 10.1371/journal.pone.0004384

**Published:** 2009-02-06

**Authors:** Angelika Banzhoff, Roberto Gasparini, Franco Laghi-Pasini, Tommaso Staniscia, Paolo Durando, Emanuele Montomoli, Pamela Capecchi, Pamela di Giovanni, Laura Sticchi, Chiara Gentile, Anke Hilbert, Volker Brauer, Sandrine Tilman, Audino Podda

**Affiliations:** 1 Novartis Vaccines, Marburg, Germany; 2 Department of Health Sciences, Section of Hygiene and Preventive Medicine, University of Genoa, Genoa, Italy; 3 Department of Allergology and Clinical Immunology, ‘Le Scotte’ University Hospital, University of Siena, Siena, Italy; 4 Department of Medicine and Aging, University G. D'Annunzio, Chieti, Italy; 5 Department of Pathophysiology, Experimental Medicine and Public Health, Laboratory of Molecular Epidemiology, University of Siena, Siena, Italy; 6 Department of Drug Sciences, University G. D'Annunzio, Chieti, Italy; 7 Novartis Vaccines, Amsterdam, The Netherlands; 8 Novartis Vaccines, Siena, Italy; Karolinska Institutet, Sweden

## Abstract

**Background:**

Pathogenic avian influenza virus (H5N1) has the potential to cause a major global pandemic in humans. Safe and effective vaccines that induce immunologic memory and broad heterotypic response are needed.

**Methods and Findings:**

Healthy adults aged 18–60 and >60 years (n = 313 and n = 173, respectively) were randomized (1∶1) to receive two primary and one booster injection of 7.5 μg or 15 μg doses of a subunit MF59-adjuvanted H5N1 (A/Vietnam/1194/2004) (clade 1) vaccine. Safety was monitored until 6 months after booster. Immunogenicity was assessed by hemagglutination inhibition (HI), single radial hemolysis (SRH) and microneutralization assays (MN). Mild injection-site pain was the most common adverse reaction. No serious adverse events relating to the vaccine were reported. The humoral immune responses to 7.5 μg and 15 μg doses were comparable. The rates for seroprotection (HI>40; SRH>25mm^2^; MN ≥40) after the primary vaccination ranged 72–87%. Six months after primary vaccination with the 7.5 μg dose, 18% and 21% of non-elderly and elderly adults were seroprotected; rates increased to 90% and 84%, respectively, after the booster vaccination. In the 15 μg group, seroprotection rates among non-elderly and elderly adults increased from 25% and 62% after primary vaccination to 92% and 88% after booster vaccination, respectively. A heterologous immune response to the H5N1/turkey/Turkey/05 strain was elicited after second and booster vaccinations.

**Conclusions:**

Both formulations of MF59-adjuvanted influenza H5N1 vaccine were well tolerated. The European Union requirement for licensure for pre-pandemic vaccines was met by the lower dose tested. The presence of cross-reactive antibodies to a clade 2 heterologous strain demonstrates that this vaccine may be appropriate for pre-pandemic programs.

**Trial Registration:**

ClinicalTrials.gov NCT00311480

## Introduction

The highly pathogenic avian influenza H5N1 virus, first reported in China in 1996, is responsible for severe avian influenza outbreaks [1]–[3]. The disease is now widespread among poultry and migratory birds in many parts of the world, and more than 380 humans have been infected, with approximately 240 (63%) deaths [4]. Based on the number of human infections, the H5N1 virus is considered the most likely candidate to cause the next pandemic [5], which is expected to spread quickly and result in substantial global morbidity and mortality [6]–[7].

Since future pandemic virus strains cannot be clearly anticipated, vaccines using strains with pandemic potential, such as H5N1, that induce immunologic memory and cross-reactivity, could form the first line of defense [8]. Due to the rapid spread and significant logistic challenges in supplying sufficient quantities of pandemic vaccine [9], [10], proactive priming of selected populations with an H5N1 pre-pandemic vaccine should be considered now.

Immunogenicity data on conventional non-adjuvanted H5N1 vaccines are not encouraging. A previous study showed that two vaccinations with 90 μg hemagglutinin (HA) of a non-adjuvanted vaccine induced an antibody response at protective levels in only half of an immunologically naïve population [11]. One study found that two 30 μg doses of an alum-adjuvanted split-virion H5N1 vaccine were needed to induce an immune response that met two of three criteria for European Union licensure [12]. Since the amount of antigen in both these cases is substantially more than is needed for protection against seasonal influenza strains, and given current limits on worldwide vaccine production capacity, measures to increase the immune response and reduce the antigen content are essential. This is particularly important as clinical trials of H5N1 vaccines have shown that two doses of adjuvanted vaccine are necessary to satisfy all European regulatory criteria for immunogenicity [12]–[14].

The use of adjuvants in vaccines is an established method for increasing the immune response and cross-reactivity and reducing the antigen content [15]. MF59^®^ is the first oil-in-water emulsion licensed as an adjuvant for human use [15] and has been shown to increase the immune response against homologous and heterologous interpandemic seasonal influenza vaccine strains in the elderly [16]–[19] and other at-risk populations [20]–[22]. The safety database for MF59 is larger and more extensive than that for any other adjuvanted influenza vaccine.

With the exception of the virus strain and the amount of antigen, the MF59-adjuvanted H5N1 vaccine used in this trial and the licensed seasonal influenza vaccine, Fluad^®^, are identical. Previous clinical trials using other potential pandemic influenza strains such as H5N3 [2], [23]–[24] and H9N2 [25] have shown that the addition of MF59: i) significantly increases the immunogenicity of the H5 and H9 antigens, ii) confers cross-reactivity against antigenically drifted strains [17], [24], and iii) allows reduction of the antigen content. This is the first study to investigate the cross-reactivity, immunogenicity and safety of an H5N1 vaccine in both non-elderly (aged 18–60 years) and elderly (aged>60 years) adult populations. In this clinical trial, the humoral immune response to two primary doses and, in a subgroup, a booster dose of a subunit MF59-adjuvanted H5N1 influenza vaccine is reported.

## Methods

### Study design and objectives

The protocol for this trial and supporting CONSORT checklist are available as supporting information; see Checklist S1 and Protocol S1This observer-blind, randomized study was conducted at four sites in Italy between March 2006 and July 2007. The primary objective was to assess the immunogenicity and safety of up to three 0.5 mL intramuscular injections of 7.5 μg or 15 μg MF59-adjuvanted H5N1 vaccine according to the regulatory guideline [26]. The study was powered for a secondary objective to statistically distinguish between primary vaccination with the 7.5 μg and 15 μg dose.

#### Participants

Healthy adults 18 years of age and above were eligible to participate in the study. Principal criteria for exclusion from the study were history of anaphylactic shock; allergy to eggs or any vaccine component; immunodeficiency or immunosuppressive therapy; participation in another clinical trial; previous receipt of an H5N1, H3 or H9 vaccine; current acute febrile disease; current use of antibiotics or antivirals; planned surgery during the study period; and, for women, pregnancy or refusal to use reliable contraception. The study was approved by the Ethics Review Committee of each participating center and was conducted in accordance with the principles of the Declaration of Helsinki and Good Clinical Practice. The study is registered in the National Institutes of Health ClinicalTrials.gov register (NCT00311480). Written informed consent was obtained from each eligible volunteer before enrollment.

#### Vaccines and randomization

Eligible participants were randomized at a 1∶1 ratio, stratified by age group, to receive two vaccinations of either 7.5 μg or 15 μg HA H5N1 (A/Vietnam/1194/2004; NIBRG-14) inactivated subunit influenza virus vaccine adjuvanted with MF59. A subgroup of the first 50% of participants enrolled at each site also received a booster vaccination with the same dose as that used for primary vaccination. Both formulations were supplied in pre-filled syringes (0.5 mL) and administered into the deltoid muscle by an un-blinded individual who had sole access to the randomization code at each site. All other site personnel and participants were blinded to the vaccine group assignment. The first two vaccinations were administered 21 days apart, and the third (booster) vaccination was administered 6 months later.

#### Safety surveillance

Each participant was observed for 30 minutes post-injection for anaphylactic reactions, and was instructed to complete a diary card on the day of vaccination and each of the 6 subsequent days to report solicited local reactions at the site of injection (i.e., ecchymosis, redness, induration, swelling and pain) and systemic reactions (i.e., chills, malaise, myalgia, arthralgia, nausea, headache, sweating, fatigue and fever). Severe solicited reactions were defined as injection-site reactions >50 mm diameter, reactions preventing the performance of normal daily activities or body temperature ≥40°C. All unsolicited adverse events were recorded for 3 weeks after each vaccination and assessed by the investigator for severity, seriousness and relationship to the study vaccine. In addition, all adverse events necessitating a physician's visit or leading to premature study discontinuation, and all serious adverse events were recorded during the 6-month follow-up periods after the second and booster vaccinations.

#### Laboratory methods

Blood samples were collected from study participants at baseline before the first vaccination, before the second vaccination, and at 3 weeks and 6 months after the second vaccination (pre-booster). In the booster subset, blood samples were also collected 3 weeks and 6 months post-booster. The modified hemagglutinin-inhibition (HI) test using horse erythrocytes was performed in the laboratory of Clinical Serology, Novartis Vaccines, Marburg, Germany and was based on the method of Stephenson and colleagues [27]. The HI titer is expressed as the reciprocal of the highest dilution at which hemagglutination was still completely inhibited. Single radial hemolysis (SRH) was based on a modified method of that devised by Schild and colleagues [28]–[29]. The European Agency for Evaluation of Medicinal Products criterion of serologic response to influenza vaccines, a SRH value of >25 mm^2^, was used as a cut-off for serologic protection [26]. Microneutralization (MN) was performed according to the method previously described by Nicholson and colleagues [2]. Serial serum dilutions started at 1∶20. The reciprocals of twofold dilutions of serum that achieved 50% or greater neutralization of virus growth were considered positive. Neutralizing titers that confer seroprotection against H5N1 influenza strains have not been agreed, but we have used a titer of ≥40, which is in line with the cut-off used in other studies [11]–[14], [23]. Control sera included human and sheep sera immunized with the influenza H5N1/Vietnam/1194/2004 strain. SRH and MN assays were performed using the homologous H5N1/Vietnam/1194/2004 strain (NIBRG-14; clade 1) and also the H5N1/turkey/Turkey/1/05 (NIBRG-23; clade 2) heterologous strain to test for cross-reactivity. Laboratory staff were blinded to the participants' vaccine dosages.

#### Statistical analyses

The planned sample size of 460 individuals (i.e., 230 subjects per study arm) was sufficient to demonstrate that the immune response to primary vaccination with 7.5 μg HA was non-inferior to the 15 μg HA formulation. For each vaccine group within each age cohort and at each blood sampling time point, the proportion of subjects seroprotected, defined as an SRH area ≥25 mm^2^ or MN titer ≥40, were calculated and two-sided 95% confidence intervals (CIs) were determined using the Clopper-Pearson method. MN titers below the detection limit (1∶20) were arbitrarily assigned to half that limit for the purpose of the analysis. Undetectable SRH areas were assigned an area of 4 mm^2^. The immune response elicited by two vaccinations formulated with 7.5 μg HA was considered non-inferior to that containing 15 μg HA if the lower limit of the two-sided 95% CI around the ratio of geometric mean titer (GMT) at 3 weeks after the second injection (GMT_7.5_/GMT_15_) exceeded 0.5. Participants were excluded from the immunogenicity analysis if they did not receive all designated vaccinations or if the planned blood collections were missed or performed outside the specified time window. Safety was analyzed in participants who received at least one vaccination and reported solicited or unsolicited adverse events.

## Results

### Study population

**Figure 1:**
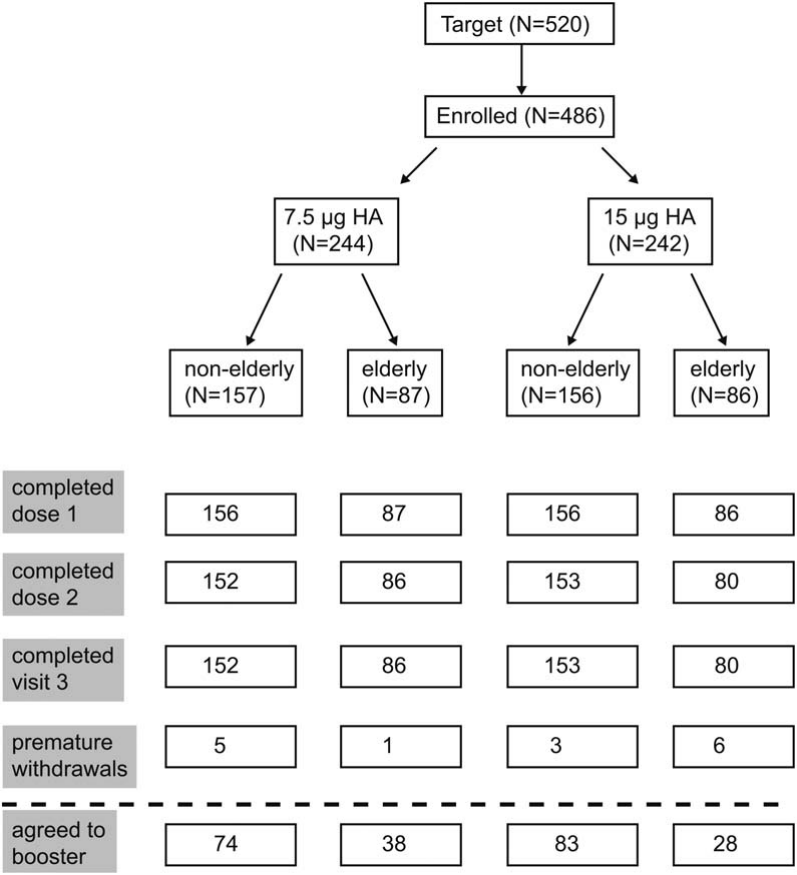
Subject disposition. A total of 486 healthy adults were enrolled in the study and randomized in a 1∶1 ratio, stratified by age group (non-elderly adults, aged 18–60 years, and elderly adults, aged >60 years) to receive two vaccinations of either 7.5 μg or 15 μg HA H5N1 (A/Vietnam/1194/2004; NIBRG-14) inactivated subunit influenza virus vaccine adjuvanted with MF59. The first two vaccinations were administered 21 days apart, and a subset of the first participants also received a third (booster) vaccination 6 months later.

The target was to enrol and randomize 520 subjects to treatment, but due to slow recruitment a total of 486 individuals were enrolled in the study, of whom 313 were 18–60 years of age and 173 were >60 years of age (Figure 1). One participant (in the 18–60 year age cohort) presented with a transient syncopal event and withdrew before receiving any vaccination. A total of 485 participants received the first vaccination and were therefore included in the safety analysis, while 471 participants (97%) received a second vaccination, and a subset of 223 participants (46%) agreed to receive a third (booster) vaccination. The booster subset was not randomized: subjects were able to accept or decline a booster dose, which led to small differences in numbers between the groups. There were no major protocol deviations and, therefore, intention-to-treat analyses are reported throughout. Within each age group, demographic characteristics were similar between the 7.5 μg and 15 μg groups (Table 1).

**Table 1:** Age, sex and ethnic origin distribution across groups

	18–60 years		>60 years	
	7.5 μg HA	15 μg HA	7.5 μg HA	15 μg HA
**Number randomized**	157	156	87	86
**Age, years Mean (range)**	43.4 (18–60)	42.3 (18–60)	71 (62–88)	70.1 (61–90)
**Ethnic origin Caucasian, %**	99	97	100	100
**Gender Male: Female, %**	45∶55	45∶55	60∶40	56∶44
**Previous influenza vaccination, %**	52	56	89	85

HA, hemagglutinin

### Safety analysis

**Figure 2:**
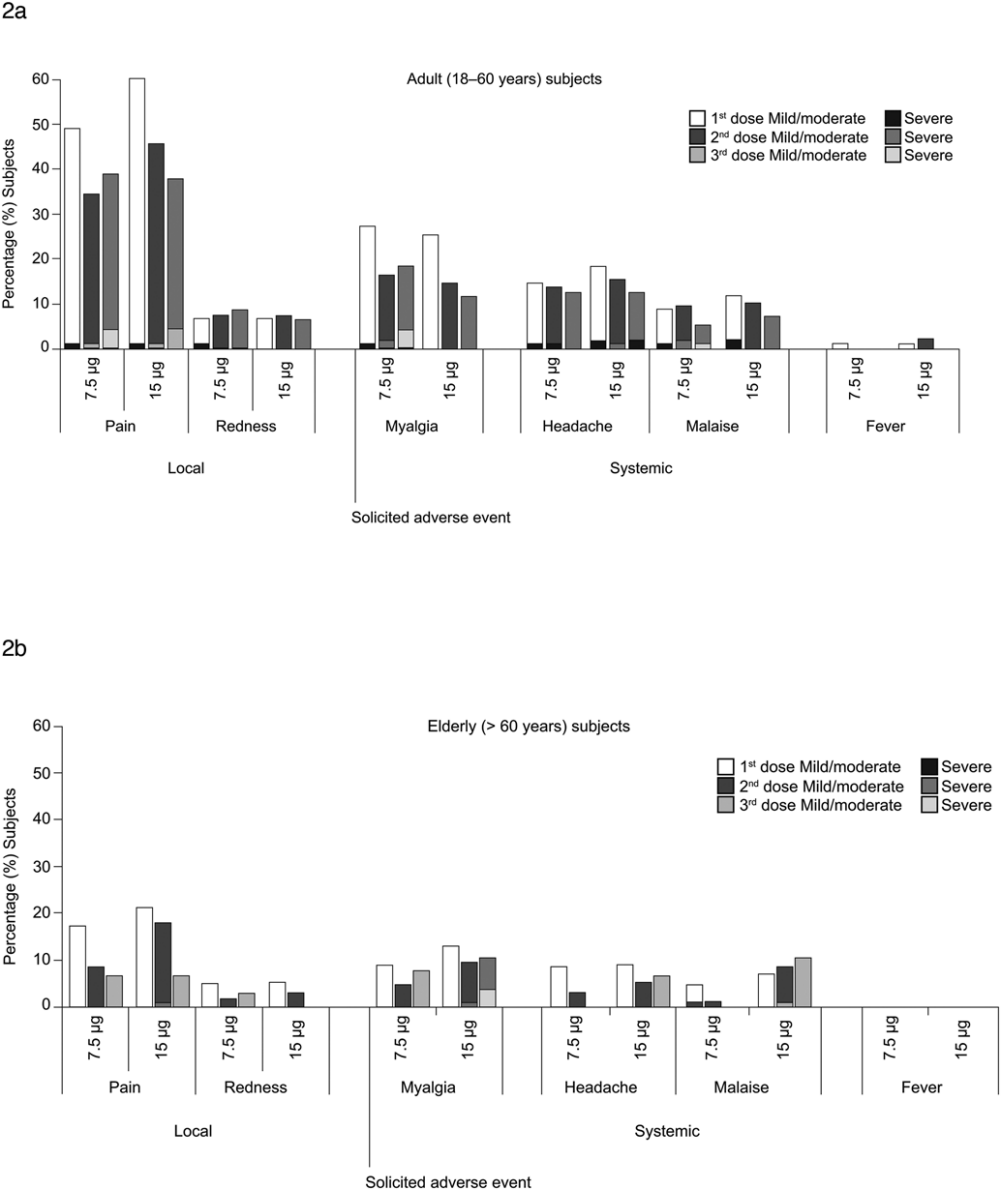
Most commonly reported solicited systemic adverse reactions. Classified as mild/moderate (white bars) or severe (grey bars) after the first, second and third vaccination in non-elderly adults (18–60 years; part a) and in elderly adults (>60 years; part b).

Both the 7.5 μg and 15 μg formulations were well tolerated, and there were no reports of serious adverse events related to the study vaccine during the study period or during follow-up (mean 13 months) (Figure 2a–b). Although injection-site pain was the most commonly reported reaction, only 2% of reported cases were severe enough to interfere with daily activities. Other severe local reactions occurred in less than 1% of the total population and were of short duration. Solicited adverse reactions were more common in adults <60 years (Figure 2a) than in the elderly population (Figure 2b); in both age cohorts their frequency decreased between the first and second vaccinations and remained stable after the third vaccination.

The most commonly reported solicited systemic adverse reactions in both age cohorts were myalgia and headache, with the highest rates occurring after the first vaccination (in the 7.5 μg group: myalgia in 28% of non-elderly and 8% of elderly and headache in 15% of non-elderly and 9% of elderly; in the 15 μg group: myalgia in 26% non-elderly and 13% elderly and headache in 19% non-elderly and 9% elderly). Severe solicited reactions were reported by less than 2% of non-elderly adults and 1% of the elderly participants (Figure 2a–b).

After primary vaccination, an axillary temperature of 38°C or greater was reported by 1% and 3% of non-elderly adults in the 7.5 μg and 15 μg groups, respectively, while no elderly participants reported fever. No participants reported severe fever (≥40°C) and no participant of any age reported fever after the booster vaccination. Fewer than 3% of individuals in either dose group reported mild to moderate non-solicited adverse events that were possibly related to the vaccine.

During the 3-week to 6-month follow-up period after the second vaccination, fewer than 3% of participants reported adverse events. One participant (18–60 years) in the 7.5 μg group reported a non-solicited adverse event possibly related to vaccination (generalized myalgia and arthralgia of moderate severity 30 days after the second vaccination). One pregnancy occurred before the second vaccination and the participant withdrew from the study and subsequently delivered a healthy full-term infant.

After the third vaccination, one non-elderly adult (1%) and one elderly (3%) participant in the 7.5 μg group and two (2%) non-elderly adults and one (4%) elderly participant in the 15 μg group reported non-solicited adverse events, all judged as unrelated to the study vaccine, during the 3-week to 6-month follow-up period. No vaccine-related serious adverse events were reported during the entire study period.

### Immunogenicity

A total of 466 of the 486 enrolled individuals were included in the immunogenicity analyses.

#### i) Primary vaccination

At baseline in both dose groups, HI titers, SRH areas and MN titers, respectively, were detectable in 0–3%, 5–9% and 3% of non-elderly adults and 11–12%, 11–24% and 15–18% of elderly participants (Tables 2, 3, 4). Elderly subjects had higher baseline antibody titers to H5N1 than younger adults. Antibody responses against H5N1 were already observed in both vaccine groups 21 days after the first vaccination, as demonstrated by HI, SRH and MN assays (Table 2, 3, 4). After the second vaccination, geometric mean ratios (GMRs) were comparable or higher in the 7.5 μg group than in the 15 μg group and also higher in non-elderly adults than in the elderly.

**Table 2:** Hemagglutination inhibition response: MF59-adjuvanted H5N1 subunit influenza vaccine formulations and age cohort

	18–60 years		>60 years	
	7.5 μg HA	15 μg HA	7.5 μg HA	15 μg HA
	n = 151	n = 147	n = 81	n = 74
**Baseline**				
GMT	5.1 (4.8–5.4)	5.5 (5.2–5.9)	8.1 (6.3–11)	8.2 (6.3–11)
Seroprotection rate, %	0 (0–2)	3 (1–7)	12 (6–22)	11(5–20)
**3 weeks after first vaccination**				
GMT	17 (13–23)	21 (16–28)	31 (20–49)	40 (25–63)
GMR	3.4 (2.6–4.5)	3.8 (2.8–5.0)	3.9 (2.6–5.7)	4.9 (3.3–7.3)
Seroprotection rate, %	34 (26–42)	39 (31–47)	51 (39–62)	53 (41–64)
Seroconversion rate, %	34 (26–42)	35 (28–44)	38 (28–50)	46 (34–58)
**3 weeks after second vaccination**				
GMT	82 (62–110)	85 (64–110)	77 (53–114)	82 (55–123)
GMR	16 (12–21)	15 (12–21)	9.52 (6.6–1.4)	10 (6.8–1.5)
Seroprotection rate, %	73 (65–80)	72 (64–79)	75 (64–84)	76(64–85)
Seroconversion rate, %	73 (65–80)	69 (61–77)	67 (55–77)	70 (59–80)
**Pre-booster (subset)**	**n = 71**	**n = 82**	**n = 37**	**n = 26**
GMT	12 (9.0–17)	16 (12–21)	26 (13–49)	42 (21–85)
Seroprotection rate, %	27 (17–39)	34 (24–45)	54 (37–71)	62 (41–80)
**3 weeks post-booster**				
GMT	138 (93–205)	104 (72–151)	129 (83–201)	212 (130–345)
GMR to pre-booster	11 (7.6–16)	6.54 (4.6–9.3)	5.02 (2.8–9.0)	5.07 (237–9.6)
Seroprotection rate, %	83 (72–91)	76 (65–84)	92 (78–98)	96 (80–100)
Seroconversion rate, %	73 (61–83)	62 (51–73)	51 (34–68)	54 (33–73)
**6 months post-booster**	**n = 69**	**n = 76**	**n = 35**	**n = 22**
GMT	27 (18–41)	29 (20–42)	36 (20–68)	71 (34–145)
Seroprotection rate, %	52 (40–64)	46 (35–58)	57 (39–74)	77 (55–92)

Numbers in parenthesis are two-sided 95% confidence intervals; Seroprotection = HI titer ≥40; Seroconversion = negative pre-vaccination serum (i.e., HI titer <10) and post-vaccination HI titer ≥40 or significant increase (at least a fourfold increase in HI titer in subjects who were positive pre-vaccination, i.e., HI titer ≥10); HA, hemagglutinin; GMT, geometric mean titer; GMR, the geometric mean of the ratio over baseline.

**Table 3:** Single radial hemolysis (SRH) response: MF59-adjuvanted H5N1 subunit influenza vaccine formulations and age cohort

	18–60 years		>60 years	
	7.5 μg HA n = 149	15 μg HA n = 149	7.5 μg HA n = 84	15 μg HA n = 80
**Baseline**				
GMA	4.8 (4.3−5.3)	5.2 (4.7−5.8)	6.0 (4.9−7.4)	7.6 (6.2−9.5)
Seroprotection rate, %	5 (2−10)	9 (5−14)	11 (5−19)	24 (15−35)
**3 weeks after first vaccination**				
GMA	11 (9.5−14)	15 (12−18)	17 (13−22)	19 (14−25)
GMR	2.4 (2.0−2.9)	2.8 (2.4−3.4)	2.9 (2.2−3.7)	2.5 (1.9−3.2)
Seroprotection rate, %	40 (32−49)	51 (43−59)	52 (41−63)	58 (46−68)
Seroconversion rate, %	38 (30–47)	42 (34–51)	44 (33–55)	43 (32–54)
**3 weeks after second vaccination**				
GMA	37 (32−43)	36 (31−41)	30 (24−37)	31 (25−39)
GMR	7.7 (6.6−9.1)	6.9 (5.9−8.0)	5.0 (3.9−6.4)	4.1 (3.2−5.3)
Seroprotection rate, %	85 (79−91)	85 (79−91)	80 (70−88)	81 (71−89)
Seroconversion rate, %	85 (78–90)	80 (73–86)	70 (59–80)	69 (57–79)
**Pre-booster (subset)**	**n = 71**	**n = 83**	**n = 38**	**n = 26**
GMA	6.8 (5.4−8.6)	8.0 (6.5−9.9)	6.3 (4.3−9.1)	16 (10−24)
Seroprotection rate, %	18 (10−29)	25 (16−36)	21 (10−37)	62 (41−80)
**3 weeks post-booster**				
GMA	41 (34−48)	42 (36−49)	32 (23−46)	42 (29−62)
GMR to pre-booster	6.0 (4.7−7.5)	5.2 (4.2−6.5)	5.2 (3.5−7.7)	2.7 (1.7−4.2)
Seroprotection rate, %	89 (79−95)	92 (83−97)	84 (69−94)	88 (70−98)
Seroconversion rate, %	83 (72–91)	81 (71–89)	63 (46–78)	65 (44–83)
**6 months post-booster**	**n = 69**	**n = 76**	**n = 35**	**n = 22**
GMA	17 (13−21)	17 (14−21)	13 (8.4−19)	25 (16−39)
Seroprotection rate, %	55 (43−67)	55 (43−67)	43 (26−61)	77 (55−92)

Numbers in parenthesis are two-sided 95% confidence intervals; Seroprotection = SRH ≥25mm^2^; Seroconversion = negative pre-vaccination serum (i.e., SRH titer ≤ 4mm^2^) and post-vaccination SRH area ≥25 mm^2^ or significant increase (at least a 50% increase in SRH area in subjects who were positive pre-vaccination, i.e., SRH area >4mm^2^); HA, hemagglutinin; GMA, geometric mean area; GMR, the geometric mean of the ratio over baseline

**Table 4:** Microneutralization response: MF59-adjuvanted H5N1 subunit influenza vaccine formulations and age cohort

	18–60 years		>60 years	
	7.5 μg HA n = 151	15 μg HA n = 151	7.5 μg HA n = 84	15 μg HA n = 80
**Baseline**				
GMT	11 (10−12)	11 (10−12)	18 (14−22)	16 (13−20)
MN ≥1∶40, %	3 (1−7)	3 (1−7)	18 (10−28)	15 (8−25)
**3 weeks after first vaccination**				
GMT	26 (22−32)	35 (29−42)	42 (31−59)	51 (37–71)
MN ≥1∶40, %	33 (26−41)	44 (36−52)	49 (38−60)	51 (40−63)
**3 weeks after second vaccination**				
GMT	117 (98−140)	102 (85−122)	80 (61−105)	90 (68−119)
MN ≥1∶40, %	85 (78−90)	81 (74−87)	79 (68−87)	76 (65−85)
**Pre-booster (subset)**	**n = 71**	**n = 83**	**n = 38**	**n = 26**
GMT	33 (26−42)	43 (34−54)	31 (20−47)	63 (39−101)
MN ≥1∶40, %	41 (29−53)	53 (42−64)	42 (26−59)	69 (48−86)
**3 weeks post-booster**				
GMT	210 (168−262)	214 (174−263)	154 (105−227)	225 (146−345)
MN ≥1∶40, %	94 (86−98)	96 (90−99)	97 (86−100)	96 (80−100)
**6 months post-booster**	**n = 69**	**n = 76**	**n = 35**	**n = 22**
GMT	34 (26−44)	35 (27−44)	25 (17−39)	46 (28−75)
MN ≥1∶40, %	41 (29−53)	46 (35−58)	37 (21−55)	64 (41−83)

Numbers in parenthesis are two-sided 95% confidence intervals; HA, hemagglutinin; GMT, geometric mean titer

**Figure 3:**
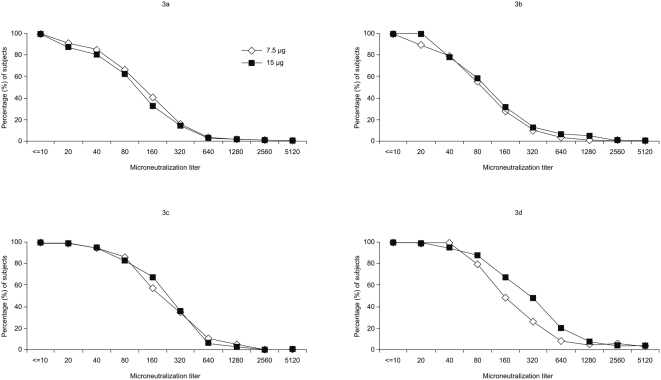
Reverse cumulative distribution curves for microneutralization antibody titers. Measured 3 weeks after a second injection and 3 weeks after a booster dose in non-elderly adults (18–60 years; parts a and c, respectively) and in elderly adults (>60 years; parts b and d, respectively).

Following two vaccinations with either formulation, 81–86% of non-elderly adults and 76–79% of elderly adults had neutralizing antibody titers of at least 40 (Figure 3a,b), and this increased to 94–96% of non-elderly adults and 96–97% of elderly adults after the booster vaccination (Figure 3c,d). These percentages were comparable with those for seroprotection rates assessed by HI and SRH (Table 2 and 3), with good Pearson correlation on log_10_-transformed titers between the assays across injection, age group and formulation (r = 0.67).

After primary vaccination (two-dose regimen) both the 7.5 μg and the 15 μg MF59-adjuvanted vaccine formulations met all the Committee for Medicinal Products for Human Use criteria for evaluation of influenza vaccines (HI and SRH assays) in both non-elderly and elderly participants [26].

#### ii) Booster vaccination

Seroprotection rates assessed by HI assay increased from 27% (18–60 years) and 54% (>60 years) immediately before the 7.5 μg booster dose to 83% and 92%, respectively, 3 weeks following the booster dose. Similar increases in seroprotection rates were observed in the 15 μg group, rising from 34% (18–60 years) and 62% (>60 years) before the booster dose to 76% and 96%, respectively, after the booster dose (Table 2). Similarly, seroprotection rates assessed by SRH increased from 18% (18–60 years) and 21% (>60 years) immediately before the 7.5 μg booster to 89% and 84%, respectively, 3 weeks following the booster (Table 3). Similar increases in seroprotection rates were observed in the 15 μg group, rising from 25% and 62% before the booster to 92% and 88% in those aged 18–60 years and >60 years, respectively. MN titers in non-elderly and elderly recipients increased from 41% to 94% and from 42% to 97%, respectively, after the 7.5 μg booster and from 53% to 96% and from 69% to 96%, respectively, after the 15 μg booster (Table 4).

### Cross-protection

An antibody response against a heterologous clade 2 strain (H5N1/turkey/Turkey/1/05) was detectable by HI assay, with cross-immunogenicity to the clade 2 strain increasing from 28–36% after the second dose to 59–70% after the third dose, across dosage groups and age cohorts. Seroprotection rates as measured by SRH were 57–77% and 78–88% after the second and third doses, respectively. The MN GMTs ranged 12–19 after the second vaccination and 44–93 after the booster for the clade 2 strain (Table 5), versus 80–117 and 154–225 for the clade 1 strain (Table 4) across formulations and age cohorts. SRH GMAs were 15–24 and 24–34 for clade 2 (Table 5) versus 30–37 and 32–42 for the clade 1 strain after primary and booster vaccination (Table 3).

**Table 5:** Heterologous response (A/turkey/Turkey/1/05 − NIBRG-23) following immunisation with MF59-adjuvanted H5N1 subunit influenza vaccine

			Assessment Parameter	7.5 μg HA	15 μg HA
**Adult**	**HI**			**n = 70**	**n = 79**
		Post-second injection (day 43)	GMT	18 (13–24) (n = 69)	14 (10–19) (n = 78)
		Seroprotection rate	%	36 (25–49)	28 (19–40)
		Post-booster injection (day 223)	GMT	58 (39–86)	46 (31–67)
		Seroprotection rate	%	70 (58–80)	59 (48–70)
	**MN**			**n = 70**	**n = 81**
		Post-second injection (day 43)	GMT	19 (15–24)	19 (15–24)
		Titer ≥40	%	27 (17–39)	21 (13–31)
		Post-booster injection (day 223)	GMT	77 (60–100)	93 (74–118)
		Titer ≥40	%	73 (61–83)	79 (69–87)
	**SRH**			**n = 70**	**n = 81**
		Post-second injection (day 43)	GMA	23 (19–28)	24 (20–28)
		Seroprotection rate	%	70 (58–80)	70 (59–80)
		Post-booster injection (day 223)	GMA	30 (26–36)	34 (29–39)
		Seroprotection rate	%	83 (72–91)	88 (78–94)
**Elderly**	**HI**			**n = 36**	**n = 26**
		Post-second injection (day 43)	GMT	14 (8.4–22)	13 (7.74–23) (N = 23)
		Seroprotection rate	%	36 (21–54)	35 (16–57)
		Post-booster injection (day 223)	GMT	36 (19–68)	49 (25–97)
		Seroprotection rate	%	67 (49–81)	65 (44–83)
	**MN**			**n = 37**	**n = 26**
		Post-second injection (day 43)	GMT	12 (9.4–16)	17 (13–23)
		Titer ≥40	%	11 (3–25)	31 (14–52)
		Post-booster injection (day 223)	GMT	44 (29–67)	61 (39–95)
		Titer ≥40	%	62 (45–78)	65 (44–83)
	**SRH**			**n = 37**	**n = 26**
		Post-second injection (day 43)	GMA	15 (9.91–22)	24 (16–36)
		Seroprotection rate	%	57 (39–73)	77 (56–91)
		Post-booster injection (day 223)	GMA	24 (17–34)	33 (23–48)
		Seroprotection rate	%	78 (62–90)	88 (70–98)

Numbers in parenthesis are two-sided 95% confidence intervals; GMT/GMA, geometric mean titer/geometric mean area ;Seroprotection rate = SRH ≥25mm^2^ or MN ≥40.

## Discussion

The H5N1 virus is the most likely candidate for an emerging pandemic strain. Owing to the anticipated rapid spread of the pandemic virus, together with the time required to produce a vaccine that matches the circulating strain, the first wave of the pandemic may have passed in many countries before a significant amount of pandemic vaccine is available. The success of national pandemic preparedness strategies may depend on providing appropriate H5N1 pre-pandemic vaccines (i) in a proactive controlled manner (ii) that induce immunologic memory and demonstrate cross-reactivity in the whole population, including the elderly, and (iii) with a proven safety profile. Evidence for the safety of the MF59 adjuvant is provided not only by the present trial, but also from a review of the database (>14,000 clinical trial participants and >30 million distributed doses post-licensure) for the interpandemic influenza vaccine Fluad^®^, which differs from the H5N1 vaccine only in its HA content and viral strain composition (2×7.5 μg or 2×15 μg H5N1 versus 1×45 μg HA seasonal viral strains). It has been well established that repeated yearly vaccination with Fluad^®^ does not lead to increased reactogenicity or other side effects [30], and the post-marketing MF59 safety database showed no increase in spontaneous reports of adverse events compared with conventional influenza vaccines. While little is published on immunopotentiators for pre-pandemic vaccines, a recent study of an H5N1 vaccine using a novel adjuvant in non-elderly adults [13] indicates a comparable immune response to that obtained with MF59. However, the novel adjuvant appeared to result in a higher incidence of mild to moderate injection-site pain [13]. In contrast, in the current study both 7.5 μg and 15 μg doses of MF59-adjuvanted H5N1 vaccine were equally well tolerated and induced neutralizing antibody responses in non-elderly and elderly adults. Long-term safety assessments of the vaccine beyond the 6-month follow-up period of this study are in progress.

As was the case in previous H5N1 vaccine trials [11]–[14], some of our study participants were not immunologically naïve at baseline. The microneutralization results in the 18–60 year age group are consistent with findings from earlier studies indicating that approximately 1–3% of subjects have H5N1 antibody at baseline [11]–[14]. The higher incidence of H5N1 antibodies in the elderly may reflect increased natural exposure or seasonal vaccination (in particular, the H1N1 component). Others have shown that approximately 34% of recipients of seasonal influenza vaccine have cross-reactive neutralizing antibody to H5N1 [31]. Since immune responses in baseline seronegative participants were similar to those in the population as a whole, the inclusion of a small number of baseline seropositive individuals does not affect the interpretation of the results.

Regardless of antigen content, the good immune response observed after two primary injections of adjuvanted H5N1 vaccine exceeded all requirements of the European Regulatory Authority [26]. More than 76% of the individuals were seroprotected or achieved an MN titer ≥40. Although correlates of protection for MN titers against H5N1 virus infections remain unknown, neutralizing antibody titers ≥40 are increasingly recognized as protective [11]–[13]. The proportion of younger adults (<60 years) with neutralizing antibody titers ≥40 after the second 7.5 μg H5N1 vaccination compared favorably with figures reported in studies using up to 90 μg of non-adjuvanted [11] or 30 μg of aluminum-adjuvanted H5N1 vaccine in individuals aged 18–40 years [12].

As expected, seroprotection rates fell significantly 6 months after primary vaccination but increased after the booster dose to levels higher than those achieved 3 weeks after the second vaccination, indicating a memory response in both elderly and non-elderly adults. As the timing of the next pandemic influenza outbreak is unknown, the memory response may be more important than persistence, as subsequent exposure to the pandemic vaccine or wild-type virus could trigger a full immunologic response.

A heterologous immune response against H5N1/turkey/Turkey/1/05 (NIBRG-23) was detectable after the primary and booster vaccinations, indicating that there is cross-reactivity between the clade 1 and clade 2 strains. Increased heterologous immune responsiveness against antigenically drifted strains presents a significant public health advantage in the event of a pandemic outbreak. We have shown elsewhere that, among primed subjects, protective cross-reactive antibody titers to diverse H5N1 virus variants can be achieved within 7 days after administration of a single dose of M59-adjuvanted vaccine [Stephenson, manuscript submitted]. In addition, the availability of a 7.5 μg formulation of MF59-adjuvanted influenza vaccine would allow a threefold increase in the number of subjects who could be vaccinated compared with seasonal trivalent influenza vaccines, enabling production of higher quantities of vaccine, in pre-pandemic and pandemic situations, as encouraged by the World Health Organization [32].

In conclusion, H5N1/Vietnam/1194/2004 influenza vaccine adjuvanted with MF59 can be safely used as a pre-pandemic vaccine. Primary vaccination of non-elderly and elderly adults induces a sufficient immune response and cross-reactivity against the clade 2 H5N1/turkey/Turkey/05 strain, and booster vaccination leads to a strong and sustained immunologic response. A low-dose antigen formulation (7.5 μg) resulted in a comparable seroprotection benefit when compared with a higher dose (15 μg). These results, in conjunction with the extensive safety data on the MF59 adjuvant, suggest that this vaccine would be a suitable choice for proactive priming in advance of pandemic influenza.

## Supporting Information

Protocol S1 Trial protocol
(0.25 MB PDF)

Checklist S1 CONSORT checklist
(0.06 MB DOC)
