# Correction: MF59^®^-Adjuvanted H5N1 Vaccine Induces Immunologic Memory and Heterotypic Antibody Responses in Non-Elderly and Elderly Adults

**DOI:** 10.1371/annotation/5f2ae0fb-53f1-48c2-aa19-9114765ba029

**Published:** 2009-03-04

**Authors:** Angelika Banzhoff, Roberto Gasparini, Franco Laghi-Pasini, Tommaso Staniscia, Paolo Durando, Emanuele Montomoli, Pier Leopoldo Capecchi, Pamela di Giovanni, Laura Sticchi, Chiara Gentile, Anke Hilbert, Volker Brauer, Sandrine Tilman, Audino Podda

The seventh author's name is incorrect. The correct name should be: Pier Leopoldo Capecchi.

