# Supplementary material for: MF59®-Adjuvanted H5N1 Vaccine Induces Immunologic Memory and Heterotypic Antibody Responses in Non-Elderly and Elderly Adults
Source: PLoS One. 2009 Feb 6;4(2):e4384. doi: 10.1371/journal.pone.0004384 (PMC2634740; doi:10.1371/journal.pone.0004384)
Supplement: Protocol S1 — Trial protocol (0.25 MB PDF) [file pone.0004384.s001.pdf]

CLINICAL STUDY PROTOCOL V87P1

Final Issued 22 DEC 05

Re-Issued 07 June 2006

Amendment N° 1

Version number 02 **Eudract number 2005-005871-14**

A Phase II, Randomized, Controlled, Observer-blind, Multi-Center Study to Evaluate Safety and Immunogenicity of Two Doses, Administered Three Weeks Apart, and a Six Months Booster Dose of Two FLUAD-like (Surface Antigen Adjuvanted with MF59C.1) Influenza Vaccines Containing 7.5 µg or 15 µg of H5N1 Influenza Antigen, in Non-elderly Adult and Elderly Subjects

SPONSOR
Novartis Vaccines and Diagnostics S.r.l.
(formerly Chiron S.r.l.)
Via Fiorentina 1,
53100 Siena, Italy

COORDINATING INVESTIGATOR:
Roberto Gasparini, MD
Dipartimento di Scienze della Salute
Università di Genova
Via Pastore 1
16129 Genova, Italy
Phone number: +39 010 3538527

MEDICAL MONITOR
Angelika Banzhoff, MD
P.O. Box 1630
35006 Marburg, Germany
Phone number: +49 (0)6421 392681
Fax: +49 06421 394667

STUDY MONITOR
Massimo Bianchini, PhD
Phone number +39 0577 243428
Fax: +39 0577 243551

CRO
MDS Pharma Services
Centro Direzionale Milanofiori, Strada 4
Palazzo A/4, 20090 Assago (Milano), Italy
Phone number: +39 02 57514768
Fax: +39 02 57790671

For trial related **emergencies out of office hours** please contact: +49 (0)151 12641663

Property of Novartis Vaccines (formerly Chiron Vaccines)

Confidential

May not be used, divulged, published or otherwise disclosed without written consent of Novartis Vaccines (formerly Chiron Vaccines).

STUDY SYNOPSIS

TITLE OF STUDY:

A Phase II, Randomized, Controlled, Observer-blind, Multi-Center Study to Evaluate Safety and Immunogenicity of Two Doses, Administered Three Weeks Apart, and a Six Months Booster Dose of Two FLUAD-like (Surface Antigen, Adjuvanted with MF59C.1) Influenza Vaccines Containing 7.5 µg or 15 µg of H5N1 Influenza Antigen, in Non-elderly Adult and Elderly Subjects.

INVESTIGATORS:

01: Roberto Gasparini, MD;
02, 03: Francesco Schioppa, MD;
04: Franco Laghi Pasini, MD.

STUDY CENTRES:

01: Dipartimento di Scienze della Salute, Sezione Igiene e Medicina Preventiva, Università di Genova, Via Pastore, 1, 16129 Genova, Italy;

02: Dipartimento di Medicina e Scienze dell'Invecchiamento, Università G. D'Annunzio, Via dei Vestini, 66100 Chieti, Italy;

03: Ufficio di Igiene e Sanità Pubblica di Lanciano, ASL Lanciano –Vasto, Via Spaventa, 37, 66034 Lanciano, Italy.

04: Struttura Complessa di Allergologia e Immunologia Clinica, Azienda Ospedaliera Universitaria Senese, Presidio Ospedaliero delle Scotte, Strada delle Scotte, 53100 Siena, Italy.

SCIENTIFIC COORDINATOR: Emanuele Montomoli, Dipartimento di Fisiopatologia, Medicina Sperimentale e Sanità Pubblica, Laboratorio di Epidemiologia Molecolare, Università degli Studi di Siena, Via Aldo Moro, 53100 Siena, Italy.

STUDY PERIOD:

Length of Enrollment: 8 weeks (approximately)
Participation per subject: 29 weeks (approximately)
55 weeks (approximately) for 6 months booster subset of subjects

CLINICAL PHASE:

Phase II

RATIONALE:

Influenza is characterized by the occurrence of frequent, unpredictable epidemics, and much less frequent, worldwide pandemics. Epidemics arise because different strains of influenza are constantly generated through antigenic drift and individuals are less or not at all protected in some years. A pandemic is responsible for higher morbidity and mortality than an epidemic because it affects a larger proportion of the population (1). The burden of epidemics, however, is cumulatively greater than that of pandemics. A worldwide pandemic is caused by the spread of a new influenza subtype arising from antigenic shift. When such a subtype enters the population, there is no natural immunity and the new virus can easily infect exposed individuals. In the 1957 pandemic ("Asian flu"), it took 5 months for the virus to travel from China to every continent and around 9 months for the pandemic to run its course. Although the great majority of deaths in current influenza epidemics occur among the elderly, a large proportion of influenza-related deaths in the 1918-1919 (H1N1), 1957-1958 (H2N2) and 1968-1969 (H3N2) pandemics were among those under 65 years of age. Over the first decade following each pandemic, this younger group accounted for decrementally smaller proportions of deaths (2). This pattern suggests that younger persons who survive infection with a new influenza A virus subtype develop long-term protection against fatal outcomes during subsequent epidemics of the same virus. Simonsen and colleagues predicted that in the next pandemic about half the associated deaths might occur initially among those less than 65 years of age (3). Furthermore, since during the 1957-1958 and 1968-1969 pandemics around two-thirds of deaths in the under 65's were in those aged 45-64 years, it would be prudent to consider targeting those aged 45-64 years during future pandemics and possibly during the following 10 years. International travel now facilitates the spread of virus around the world. Many novel influenza virus strains arise in China and South-east Asia, which are now common business and tourist destinations, again increasing the probability of novel strains being contracted and transported.

Once a pandemic begins, it will be too late to accomplish the many key activities required to minimize the impact (4). Therefore, planning and implementation of preparatory activities must start well in advance. The World Health Organization (WHO) has provided detailed guidance on the content of such plans (5). The 56th World Health Assembly adopted a specific resolution on May 28, 2003 to ensure that all WHO Member States give priority to influenza pandemic preparedness planning (6). This includes paying particular attention to the need to ensure adequate supplies of pandemic vaccine (7). Vaccines form the main prophylactic measure against pandemic influenza and in Europe play an important role in national and EU pandemic preparedness plans. Indeed, in the event of a pandemic, a specific monovalent vaccine against the emerging strain will have to be developed rapidly, then registered and produced in very large quantities. Speed in such vaccine development is vital and Regulatory Authorities produced Guidelines to provide the basis for a fast track licensing procedure for pandemic vaccines. In the EU, according to the EMEA, this procedure involves the submission and approval of a core pandemic dossier during the inter-pandemic period, followed by a fast track approval of the pandemic vaccine, based on the submission of pandemic variation. Information in the core pandemic dossier should support a vaccination strategy that is likely to be used for a

pandemic vaccine. To achieve this, a “mock-up” vaccine should be produced, ideally in the same way as the intended pandemic vaccine and have the same antigen content and adjuvant system (if used). The variation in case of a real pandemic can be submitted with a different antigen type.

Preliminary findings have identified the H2, H5, H6, H7 and H9 subtypes of influenza A virus as those most likely to be transmitted to humans and therefore presenting a potential pandemic threat (8). When reverse genetics approach is not applied, candidate vaccines most valuable for vaccine production are considered to be A/H7N1, A/H7N7, A/H7N3 and A/H9N2 (9). Recent A/H5N1 avian influenza cases in Asia have led to the use of reverse genetics in the preparation of an H5 pandemic strain (10, 11). From 2003 until now, more than 115 human cases of H5N1 infection have been ascertained worldwide and 60 people died (more than 52% mortality rate). Virus left the original geographical region and appears today in mainland China, Philippines, Siberia and Europe. Tests conducted by the World Organisation for Animal Health (OIE) have confirmed the presence of highly pathogenic H5N1 avian influenza in samples taken from domestic birds in Turkey. In Romania, investigations of recent poultry deaths have, to date, identified the H5 subtype of avian influenza virus.

Previous clinical experience suggests that one dose of a pandemic vaccine, even in an adjuvanted formulation, is unlikely to induce a satisfactory immune response in unprimed, naïve individuals. Therefore the most likely scenario is that two doses of a pandemic vaccine will be necessary. Actually, a recent clinical trial has shown that for unadjuvanted vaccines 2 immunizations with 90 µg of strain-specific hemagglutinin (HA) are necessary to achieve protective levels of antibodies (12), which means 12 times the normal 15 µg/dose required for the inter-pandemic seasonal influenza immunization. At the same time, use of an adjuvant allows reducing the quantity of antigen per dose and would potentially lead to increased vaccine production capacities. This is the only current practical solution to address the manufacturing capacity and to effectively respond to the global need for vaccination against a pandemic influenza.

To address the need for a pandemic vaccine, Novartis (formerly Chiron) is committed to developing, registering, producing and supplying influenza pandemic vaccines. Novartis Vaccines and Diagnostic S.r.l. (formerly Chiron S.r.l.) in Siena, Italy, is producing a A/H5N1 pandemic, surface antigen, inactivated influenza vaccine, with MF59C.1 (MF59) adjuvant, to be tested in clinical trials. Chiron (now Novartis) started dealing with H5N1 in 1997 and performed the first Phase I clinical trial against a pandemic influenza virus in 1999, using its MF59 adjuvanted, egg-derived, influenza pandemic vaccine formulation with a H5N3 strain (13, 14). The pandemic FLUAD-like vaccine is a monovalent, surface antigen, inactivated influenza vaccine, adjuvanted with MF59, having as Drug Substance surface antigens from a virus strain candidate for a potential pandemic situation. The FLUAD-like vaccine contains the same adjuvant and is manufactured with the same process used for FLUAD, a Novartis (formerly Chiron) inter-pandemic, trivalent influenza vaccine. FLUAD is licensed and marketed in many European countries and also

outside Europe. The MF59 adjuvant contained in FLUAD is an oil-in-water emulsion, composed mainly of squalene, that is an intermediate metabolite in the synthesis of cholesterol. To date Chiron (now Novartis) has performed two Phase I clinical trials on the FLUAD-like influenza vaccine with two pandemic strains (H5N3 and H9N2). In both studies it was shown that two doses containing 7.5 µg of antigen are enough to reach protective levels of antibodies if the MF59 adjuvant is used. Anyway, the clinical picture is currently referred to two small size Phase I clinical trials and there is therefore the need to confirm the previous data with a statistically powered Phase II clinical trial.

The present study aims to evaluate safety and immunogenicity of two doses, administered three weeks apart, and 6 months booster of two FLUAD-like influenza vaccines containing 7.5 µg or 15 µg of H5N1 influenza antigen, in non-elderly adult and elderly subjects.

OBJECTIVES:

<u>Immunogenicity Objectives</u>

Primary

To assess immunogenicity of two 0.5 mL intramuscular (IM) injections of two FLUAD-like influenza vaccines containing either 7.5 µg or 15 µg of H5N1 influenza antigen, as measured by hemagglutination inhibition (HI) test in compliance with the requirements of the current European Union recommendations (CPMP/BWP/214/96).

Secondary

To compare immunogenicity of two 0.5 mL IM injections of two FLUAD-like influenza vaccines containing either 7.5 µg or 15 µg of H5N1 influenza antigen in terms of post-immunization geometric mean titers (GMTs) as measured by HI test.

To evaluate immunogenicity of one and three 0.5 mL IM injection of two FLUAD-like influenza vaccines containing either 7.5 µg or 15 µg of H5N1 influenza antigen, as measured by HI test.

To evaluate immunogenicity after one, two and three 0.5 mL IM injections of two FLUAD-like influenza vaccines containing either 7.5 µg or 15 µg of H5N1 influenza antigen, as measured by microneutralization (MN) test and possibly Single Radial Hemolysis (SRH) test in a subset of subjects selected in a 1:1 ratio with respect to the study vaccines and to the age group.

<u>Safety Objectives</u>

To evaluate the safety of the administration of three 0.5 mL IM injections of two FLUAD-like influenza vaccines containing either 7.5 µg or 15 µg of H5N1 influenza antigen.

METHODS:

In this phase II, randomized, controlled, observer-blind, multi-center study, subjects will be enrolled into two groups according to age (18-60 years and 61 years and over) and randomly assigned in a 1:1 ratio to one of the following vaccine groups:

Group A – two 0.5 mL injections, three weeks apart, of FLUAD-like influenza vaccine containing 7.5 µg of H5N1 influenza antigen, plus 0.5 mL booster injection at day 202 in, at least, the first half of the subjects enrolled in this group at each study site.

Group B – two 0.5 mL injections, three weeks apart, of FLUAD-like influenza vaccine containing 15 µg of H5N1 influenza antigen, plus 0.5 mL booster injection at day 202 in, at least, the first half of the subjects enrolled in this group at each study site.

Subjects will be observed for 30 minutes after each injection for any immediate reactions. All subjects will be instructed to complete a diary card to record local (i.e., ecchymosis, erythema, induration, swelling and pain at injection site) and systemic reactions (i.e., chills, malaise, myalgia, arthralgia, nausea, headache, sweating, fatigue and potential indicators of oculo-respiratory syndrome) and axillary temperature starting on the day of vaccination (after 6 hours) and for each of the 6 days following each immunization.

All adverse events will be collected for 3 weeks following each vaccination. All adverse events necessitating a physician's visit or consultation and/or leading to premature study discontinuation and all serious adverse events will be collected throughout the entire trial and data will be reconciled at study termination visit.

Serum samples for immunogenicity assays will be collected at day 1 (pre-immunization), at day 22, at day 43, at day 202 in all subjects and at day 223 and 382 in the booster subset. Immunogenicity will be evaluated by HI test in all subjects, and additionally by MN and possibly SRH in a subset of at least 220 subjects for each test.

All subjects enrolled in this study will be informed that they may be invited to participate to an extension study approximately 12 months after the completion of this vaccination series.

NUMBER OF SUBJECTS PLANNED:

460 evaluable subjects are necessary for this study. The number of subjects actually enrolled may exceed this number depending on the expected drop-out rate at the study

sites. All subjects enrolled in this study will be randomly assigned in a 1:1 ratio to one of the following vaccine groups:

Group A – FLUAD-like influenza vaccine containing 7.5 µg of H5N1 influenza antigen

Group B – FLUAD-like influenza vaccine containing 15 µg of H5N1 influenza antigen

Vaccine	FLUAD-like influenza vaccine (7.5 µg/dose)	FLUAD-like influenza vaccine (15 µg/dose)	Total
Total	230	230	460

The first half of the subjects enrolled in the study at each site will receive a booster immunization at day 202 with the same study vaccine assigned for the primary vaccination series.

SUBJECTS CHARACTERISTICS AND MAIN CRITERIA FOR INCLUSION/EXCLUSION:

Inclusion Criteria

Subjects eligible for enrollment into this study are male and female adult volunteers who are:

1. 18 years of age or older, mentally competent, willing and able to give written informed consent prior to study entry;
2. able to comply with all the study requirements
3. in general good health as determined by:
   - medical history
   - physical examination
   - clinical judgment of the investigator

Informed consent must be obtained for all the subjects before enrollment in the study.

Exclusion Criteria

Individuals are not to be enrolled into the study if:

1. they have any serious disease such as:
   - cancer (except for benign or localized skin cancer and non metastatic prostate cancer not presently treated with chemotherapy)
   - autoimmune disease (including rheumatoid arthritis)
   - advanced arteriosclerotic disease or complicated diabetes mellitus
   - chronic obstructive pulmonary disease (COPD) that requires oxygen therapy
   - acute or progressive hepatic disease
   - acute or progressive renal disease
   - congestive heart failure

2. they are hypersensitive to eggs, chicken protein, chicken feathers, influenza viral protein, neomycin or polymyxin or any other component of the vaccine;

3. they have a history of neurological symptoms or signs, or anaphylactic shock following administration of any vaccine

4. they have a known or suspected (or have a high risk of developing) impairment/ alteration of immune function (excluding that normally associated with advanced age) resulting, for example, from:
   - receipt of immunosuppressive therapy (any parenteral or oral corticosteroid or cancer chemotherapy/radiotherapy) within the past 60 days and for the full length of the study
   - receipt of immunostimulants
   - receipt of parenteral immunoglobulin preparation, blood products and/or plasma derivates within the past 3 months
   - suspected or known HIV infection or HIV-related disease

5. women who are pregnant, or women able to bear children but not willing to practice acceptable contraception for the duration of the trial;

6. within the past 4 weeks they have received:
   - another vaccine
   - any investigational agent

7. within the past 7 days, they have experienced:
   - any acute disease
   - infections requiring systemic antibiotic or antiviral therapy (chronic antibiotic therapy for urinary tract prophylaxis is acceptable)

8. within the past 3 days, they have experienced:
   - fever (i.e., axillary temperature ≥ 38°C);

9. they are taking part in another clinical study;

10. they have surgery planned during the study period

11. they have any condition which, in the opinion of the investigator, might interfere with the evaluation of the study objective.

TEST VACCINES, ANTIGEN CONTENT, DOSAGE REGIMEN, ROUTE OF ADMINISTRATION:

Vaccine A: FLUAD-like influenza vaccine containing 7.5 µg of the A/Vietnam/1194/2004-like (H5N1) influenza antigen**.**

Name of ingredients	Quantity per dose
**Active substances:**	
A/Vietnam/1194/2004-like (H5N1)	≥ 7.5 µg
**Excipients:**	
sodium chloride	4.00 mg
Potassium chloride	0.10 mg
Potassium dihydrogen phosphate	0.10 mg
disodium phosphate dihydrate	0.66 mg
calcium chloride dihydrate	0.06 mg
Magnesium chloride hexahydrate	0.05 mg
water for injections	up to 0.50 mL
**Adjuvants:**	
Squalene	9.75 mg
Polysorbate 80	1.175 mg
sorbitan trioleate	1.175 mg
sodium citrate	0.66 mg
citric acid	0.04 mg

Vaccine B: FLUAD-like influenza vaccine containing 15 µg of the A/Vietnam/1194/2004-like (H5N1) influenza antigen**.**

**Name of ingredients**	**Quantity per dose**
**Active substances:**	
A/Vietnam/1194/2004-like (H5N1)	≥ 15 µg
**Excipients:**	
sodium chloride	4.00 mg
Potassium chloride	0.10 mg
Potassium dihydrogen phosphate	0.10 mg
disodium phosphate dihydrate	0.66 mg
calcium chloride dihydrate	0.06 mg
Magnesium chloride hexahydrate	0.05 mg
water for injections	up to 0.50 mL
**Adjuvants:**	
Squalene	9.75 mg
polysorbate 80	1.175 mg
sorbitan trioleate	1.175 mg
sodium citrate	0.66 mg
citric acid	0.04 mg

Two 0.5 mL IM injections of either vaccine A or B, will be administered three weeks apart in the deltoid muscle of the (preferably) non-dominant arm.
One 0.5 mL injection of either vaccine A or B (as randomized on enrollment), will be administered in the deltoid muscle of the (preferably) non-dominant arm at day 202 in a subset of subjects.

REFERENCE VACCINES, ANTIGEN CONTENT, DOSAGE REGIMEN, ROUTE OF ADMINISTRATION:

None.

CONCOMITANT VACCINES:

No concomitant vaccination is permitted for the duration of the study except for post-exposure vaccination in a medical emergency, e.g., hepatitis, rabies, tetanus.

MEASURES OF IMMUNOGENICITY:

The measures of immunogenicity, collected for all evaluable subjects by using HI test and, in a subset of subjects, by using MN and possibly also SRH tests, are:

- the GMTs (GMAs) at day 1, at day 22, at day 43, at day 202, at day 223 and at day 382 as determined by HI, SRH and MN.
- the day 22/day 1, the day 43/day 22, the day 43/day 1, the day 202/day 43 and the day 202/day 1, the day 223/day 202 and the day 223/day 1, the day 382/day 223, the day 382/day 202 and the day 382/day 1 geometric mean titer ratios (GMRs) as determined by HI, SRH and MN.
- the percentage of subjects achieving seroconversion[1] or significant increase in antibody titer[2] at day 22, at day 43, at day 202, at day 223 and at day 382, as determined by HI and SRH.
- the percentage of subjects with at least a four-fold rise in titer at days 22, at day 43, at day 202, at day 223 and at day 382 as determined by HI, SRH and MN.
- the percentage of subjects achieving a HI or MN titer ≥ 40 (an SRH area ≥ 25mm^2) at day 1, at day 22, at day 43, at day 202, at day 223 and at day 382 as determined by HI, SRH and MN.

[1] Seroconversion is defined as negative pre-vaccination serum / post-vaccination titer ≥ 40 (area ≥ 25 mm^2).
[2] Significant increase in antibody titer is defined as at least a fourfold increase from non-negative pre-vaccination serum (≥ 10) (a 50% increase in area).

MEASURES OF SAFETY:

Number and percentage of subjects with at least one local reaction between 1 and 7 days after each vaccine injection.

Number and percentage of subjects with at least one systemic reaction between 1 and 7 days after each vaccine injection.

Number and percentage of subjects with at least one adverse event between day 1 and the study termination visit.

SEROLOGY:

Serum samples will be assessed by means of strain-specific HI, MN and possibly also SRH tests against H5N1 and, possibly, other strains. HI and MN tests will be performed at Novartis Vaccines, Clinical Serology Laboratory, Marburg, Germany; SRH test will be performed at Dipartimento di Fisiopatologia, Medicina Sperimentale e Sanità Pubblica, Laboratorio di Epidemiologia Molecolare, Università di Siena, Siena, Italy.

CRITERIA FOR ASSESSING IMMUNOGENICITY OBJECTIVES:

Primary immunogenicity objective:

To assess the primary immunogenicity study objective, all the following assessments should meet the CHMP requirements (by using HI assay) 3 weeks after the 2nd immunization:

- *Non-elderly adult subjects 18-60 years (i.e., ≥18 and < 61)*

- number of seroconversions[1] or significant increase in antibody titer[2] > 40%
- mean geometric increase > 2.5
- the proportion of subjects achieving an HI titer ≥ 40 should be > 70%·

- *Elderly subject 61 years and over (i.e., ≥ 61)*

- number of seroconversions[1] or significant increase in antibody titer[2] > 30%
- mean geometric increase > 2.0
- the proportion of subjects achieving an HI titer ≥ 40 should be > 60%

1 Seroconversion is defined as negative pre-vaccination serum (< 10) / post-vaccination titer ≥ 40.
2 Significant increase in antibody titer is defined as at least a fourfold increase from non-negative pre-vaccination serum (≥ 10)

Secondary immunogenicity objective:

To demonstrate non-inferiority of the antibody response elicited, as determined by using HI test, by two 0.5 mL IM injections of FLUAD-like influenza vaccine containing 7.5 µg of H5N1 influenza antigen vs. two 0.5 mL IM injections of FLUAD-like influenza vaccine containing 15 µg of H5N1 influenza antigen, in terms of post-immunization GMT of the two vaccines obtained 3 weeks after the second immunization.

To evaluate: immunogenicity of one 0.5 mL IM injection of FLUAD-like influenza vaccines containing either 7.5 µg or 15 µg of H5N1 influenza antigen at day 22; 6 months persistence at day 202 after two 0.5 mL IM injection of FLUAD-like influenza vaccines containing either 7.5 µg or 15 µg of H5N1 influenza antigen and to evaluate booster

response at day 223 and 6 months persistence of the booster at day 382, as measured by HI, in compliance with the requirements of the current European Union recommendations (CPMP/BWP/214/96), and by MN and possibly SRH (in a subset of subjects).

CRITERIA FOR ASSESSING SAFETY OBJECTIVES:

Safety will be assessed in accordance with available safety data on influenza vaccines.

STATISTICAL HYPOTHESIS

There is no statistical null hypothesis associated with the primary immunogenicity objective, which will be analyzed descriptively.

The null hypothesis for the secondary immunogenicity objective states that a regimen consisting in two doses of FLUAD-like influenza vaccine containing 7.5 µg each does not comply with the non-inferiority assumption that the lower limit of the 95% confidence interval (CI) of the post-immunization (day 43) GMT ratio is > 0.5, by using HI test, when compared to two doses of FLUAD-like influenza vaccine containing 15 µg.

H_0: $GMT_{7.5} / GMT_{15} \leq 0.5$

H_1: $GMT_{7.5} / GMT_{15} > 0.5$

STATISTICAL POWER CONSIDERATIONS

The sample-size calculation is based on the secondary immunogenicity objective of non-inferiority between two doses of FLUAD-like influenza vaccine containing 7.5 µg vs. two doses of FLUAD-like influenza vaccine containing 15 µg, as measured by HI test.

A 0.025 one-sided alpha level, a clinically relevant value of 0.5 in terms of the ratio of post-immunization GMTs (day 43, visit 3) between the two vaccine doses (i.e., a difference of 0.301 in terms of log [GMTs] between vaccines) and a power of 80% are chosen.

Assuming a standard deviation of 1.0 (calculated as the upper limit of the 80% CI of the standard deviation reported in a previous Chiron Vaccines (now Novartis Vaccines) pilot study, V7P37) for both formulations of FLUAD-like influenza vaccine, 230 evaluable subjects per group (460 in total) will be necessary to test the null hypothesis. Considering the drop-outs, a suitable number of subjects should be enrolled in order to achieve at least 460 evaluable subjects.

In total 220 enrolled subjects will be consecutively selected for immunogenicity evaluation by using MN assay and possibly SRH, in order to achieve a subset of at least 200 evaluable subjects for each test (100 per age group, and within them 50 per vaccine type).

INTERIM ANALYSIS

No interim analysis of data from this trial is planned. Should it later become necessary, the analysis will be governed by the procedures specified in the Novartis BCDM standard operating procedure entitled "Interim Analysis in a Clinical Trial".

A preliminary analysis of immunogenicity and safety will be performed when all Visit 3 results are available.

TABLE OF CONTENTS

Study Synopsis....2

Table of Contents....15

1. List of Abbreviations and Definitions of Terms....19

2. Ethics....21

2.1 Approval of Study Protocol....21

2.2 Ethical Conduct and Good Clinical Practice....21

2.3 Informed Consent of Subject and Confidentiality....21

2.3.1 Informed Consent of Subject....21

2.3.2 Subject Confidentiality....22

2.4 Liability and Insurance....23

3. Investigators and Study Administrative Structure....24

4. Introduction....25

5. Study Objectives....28

5.1 Immunogenicity Objectives....28

5.2 Safety Objectives....28

6. Overall Study Design....29

6.1 Overall Study Design and Plan – Description....29

6.1.1 Overall Study Design....29

6.1.2 Planned Duration of Study....29

6.1.3 Premature Discontinuation of the Study....30

6.1.3.1 Stopping/Pausing Rules....30

6.2 Discussion of Overall Study Design....30

6.3 Selection of Study Population 30
6.3.1 Inclusion Criteria 30
6.3.2 Exclusion Criteria 31
6.3.3 Removal of Subjects from Therapy or Assessment 32
6.4 Vaccines 33
6.4.1 Vaccines Administered 33
6.4.2 Identity of Investigational Vaccine 33
6.4.2.1 Vaccine Composition 33
6.4.2.2 Vaccine Labeling, Storage and Packaging 35
6.4.2.3 Vaccine Administration 37
6.4.3 Method of Assigning Subjects to Vaccination Groups 38
6.4.4 Selection of Doses in the Study 38
6.4.5 Blinding 38
6.4.6 Prior and Concomitant Therapy 38
6.4.7 Vaccination Compliance 39
6.4.8 Adherence to Randomization List 39
6.5 Immunogenicity and Safety Variables 39
6.5.1 Immunogenicity and Safety Measurements Assessed 39
6.5.2 Appropriateness of Measurements 40
6.5.3 Immunogenicity Variables 40
6.5.4 Safety Variables 40
6.5.4.1 Adverse Events 41
6.5.4.2 Serious Adverse Events 42
6.5.4.3 Local and Systemic Reactions 43

6.5.4.4 Clinical Laboratory Tests 43

6.5.4.5 Documentation/Monitoring/Reporting of Safety Variables.......................43

6.5.5 Study Procedures and Flowchart 45

6.5.5.1 Study Evaluations 50

6.6 Study Monitoring, Auditing and Documentation 51

6.6.1 Study Monitoring 51

6.6.2 Source Data Verification and Audits 52

6.7 Data Management 52

6.7.1 Data Handling Procedures 53

6.7.2 Data Protection..53

6.8 Changes in the Conduct of the Study or Planned Analysis 53

6.9 Statistical Methods and Determination of Sample Size.......................................54

6.9.1 Statistical Plans 54

6.9.1.1 Analysis of Demographic and Baseline Characteristics 54

6.9.1.2 Analysis of Immunogenicity Criteria...54

6.9.1.3 Analysis of Safety Criteria...56

6.9.1.4 Interim Analysis Planned...57

6.9.1.5 Preliminary Analysis..57

6.9.2 Determination of Sample Size 57

6.10 Documentation of Study Findings 58

6.11 Record Retention 59

6.12 Use of Information and Publication..59

7. Reference List 61

8. Investigator Agreement..63

9. Sponsor Agreement 65

Appendix A List of Central Laboratories 66

Appendix B Shipping and Handling of Laboratory Specimens 67

Appendix C Serology Procedures 69

1. LIST OF ABBREVIATIONS AND DEFINITIONS OF TERMS

Abbreviations

AE	adverse event
BCDM	Biostatistics and Clinical Data Management
CHMP	Committee for Medicinal Products for Human Use
CI	Confidence Interval
CPMP	Committee for Proprietary Medicinal Products
CQA	Clinical Quality Assurance
CRA	Clinical Research Associate
CRF	case report form
CRO	Contract Research Organization
DCF	Data Clarification Form
EC	ethics committee
EMEA	European Agency for the Evaluation of Medicinal Products
GCP	Good Clinical Practice
GMA	Geometric Mean Area
GMT	Geometric Mean Titer
GMR	Geometric Mean Ratio
HI	Hemagglutination Inhibition
ICH	International Conference on Harmonization
IM	Intramuscular
ITT	intention-to-treat
LSLV	Last Subject Last Visit
MD	Medical Doctor
MN	Microneutralization
PP	per protocol
SAE	serious adverse event
SOP	standard operating procedure
SRH	Single Radial Hemolysis
WHO	World Health Organization

Definitions of Terms

End of Trial: The End of Trial corresponds to the last visit of the last subject undergoing the trial (LSLV, Last Subject Last Visit).

Sponsor: An individual, company, institution, or organization which takes responsibility for the initiation, management, and/or financing of a clinical trial. Novartis Vaccines and Diagnostics S.r.l. is part of Novartis.

Adverse Event: An adverse event (AE) is any untoward medical occurrence in a patient or clinical investigation subject administered a pharmaceutical product at any dose that does not necessarily have to have a causal relationship with this treatment. An AE can,

therefore be any unfavorable and unintended sign (including an abnormal laboratory finding, for example), symptom, or disease temporally associated with the use of an investigational product, whether or not considered related to the investigational product. This definition includes intercurrent illnesses or injuries and exacerbation of pre-existing conditions.

Serious Adverse Event: Any experience or reaction that suggests a significant hazard, contraindication, side effect, or precaution. These events include any experience that is fatal or life-threatening, requires or prolongs inpatient hospitalization, is permanently disabling, leads to a congenital abnormality, requires intervention to prevent permanent impairment or damage, or is an important and significant medical event that, based upon appropriate medical judgment, may jeopardize the subject. Vaccine failure should also be considered as a serious adverse event (SAE).

Local and Systemic Reactions: Selected local and systemic AEs are routinely monitored in vaccine clinical trials as indicators of vaccine reactogenicity. It is recognized that each of these events, and particularly those of a systemic nature, may under some circumstances, in any individual subject, have a cause that is unrelated to the study vaccine. However, as a matter of convenience and in accordance with common clinical practice, all such events occurring within 7 days after immunization (including vaccination day) are herein termed “local and systemic reactions.”

Month, Day: Study months are based upon 30-day cycles. The study day refers to the number of days after enrollment, with the day of first vaccination being designated day 1.

2. ETHICS

2.1 Approval of Study Protocol

Chiron (now Novartis) or the investigator will provide the ethics committee (EC) with all appropriate material, including the informed consent document, according to the local regulation. The EC should also be asked for a written statement regarding the composition of the committee and should comply with GCP and with the applicable regulatory requirement(s). The trial will not be initiated until appropriate EC approval of the protocol and the informed consent document. In addition, all documents will be submitted to other authorities in compliance with local jurisdictions. Prior to enrollment, the sponsor and the investigator must exchange written confirmation that their ethical and legal responsibilities have been observed. The EC and, if applicable, other authorities must be informed of protocol amendments in accordance with local legal requirements. Appropriate reports on the progress of the study will be made to the EC and the sponsor by the investigator in accordance with applicable governmental regulations and in agreement with policy established by the sponsor.

2.2 Ethical Conduct and Good Clinical Practice

This trial will be conducted in accordance with the current version of the Declaration of Helsinki, Good Clinical Practice (GCP) according to International Conference on Harmonisation (ICH) guidelines, and applicable standard operating procedures (SOPs). Specifically, this trial is based on adequately performed laboratory and animal experimentation; the trial will be conducted under a protocol reviewed and approved by an EC; the trial will be conducted by scientifically and medically qualified persons; the benefits of the study are in proportion to the risks; the rights and welfare of the subjects will be respected; the physicians conducting the trial do not find the hazards to outweigh the potential benefits; each subject, or where applicable, each subject's legally acceptable representative(s) will give his or her written informed consent before any protocol-driven tests or evaluations are performed. A copy of the ICH GCP guidelines and of the Declaration of Helsinki will be included in the investigator's study file.

2.3 Informed Consent of Subject and Confidentiality

2.3.1 Informed Consent of Subject

The investigator is responsible to obtain informed consent in adherence to GCP and according to applicable regulations prior to entering the subject into the trial.

The information about the trial must be given orally and in an understandable form. Written information about the trial will also be provided. In addition to the explanation of the trial and of subject’s legal rights the information should comprise that access to original medical records and processing of coded personal information must be authorized. The informed consent discussion must be conducted by a person who is

qualified according to applicable local regulations. The subject should have the opportunity to inquire about details of the trial and to consider participation.

The informed consent form (ICF) must be signed and dated by the subject and must be countersigned by the person who conducted the informed consent discussion (according to local laws and GCP).

If a subject is unable to read or write, oral consent in the presence of an impartial witness is possible, if this is permitted by local legislation. In this case, the witness is to be present during the meeting in which the significance of the informed consent will be orally explained. After the informed consent discussion and after the subject has orally consented to participate in the clinical trial the witness should sign and personally date the consent form to attest that information concerning the clinical trial and the subject's rights was accurately explained to, and apparently understood by the subject and that informed consent was given freely.

The investigator will provide a copy of the signed informed consent to the subject, and will maintain the original in the investigator's study file.

The written informed consent form and any other written information to be provided to subjects should be revised whenever important new information becomes available that may be relevant to the subject's consent. Any revised written informed consent form, and written information should receive EC's approval before use.

The subject should be informed in a timely manner if new information becomes available that may affect the decision to participate in the clinical trial. The communication of this information should be documented.

2.3.2 Subject Confidentiality

Subject names will not be supplied to the sponsor. Only the subject numbers and subject initials (first, middle and last name) will be recorded in the case report form (CRF), and if a subject's name appears on any other document (e.g., pathologist report), it will be obliterated before the copy of the document is supplied to the sponsor. Study findings stored on a computer will be subject to local data protection laws. The subject, or where applicable, the subject's legally acceptable representative(s) will be informed that representatives of the sponsor, EC or regulatory authorities may inspect their medical records to verify the information collected, and that all personal information made available for inspection will be handled in the strictest confidence.

The investigator or designee will maintain a personal list of subject numbers and subject initials to enable records to be found at a later date.

2.4 Liability and Insurance

The involved parties will be insured, in accordance with applicable laws and regulations, against financial loss resulting from personal injury and/or other damages, which may arise as a consequence of this study. The sponsor will provide adequate insurance for the investigator according to regulatory requirement(s). If required by local law, study subjects enrolled into this clinical trial will also be insured against any injury resulting from the clinical study.

3. INVESTIGATORS AND STUDY ADMINISTRATIVE STRUCTURE

The trial will be administered and monitored by employees or representatives of Chiron (now Novartis) Vaccines. Study / Site Monitors will monitor the site on a periodic basis and perform verification of source documentation for each subject. The Medical Monitor will be readily available to provide appropriate medical expertise on trial related medical questions. Novartis (formerly Chiron) Regulatory Affairs or Pharmacovigilance department will be responsible for the timely reporting of serious adverse events (SAEs).

Coordinating Investigator

Roberto Gasparini, MD, Dipartimento di Scienze della Salute, Università di Genova, Via Pastore, 1, 16129 Genova, Italy. Phone number: +39 010 3538527.

Novartis (formerly Chiron) Staff

Medical Monitor: Angelika Banzhoff, MD, P.O. Box 1630, 35006 Marburg, Germany. Phone number: +49 6421 392681; Fax: +49 6421 394667.
Study Monitor: Massimo Bianchini, PhD, Novartis Vaccines and Diagnostics S.r.l., Via Fiorentina 1, 53100 Siena, Italy. Phone number: +39 0577 243428; Fax number: +39 0577 243551.

Biostatistician: Sandrine Tilman, MSc, Novartis BCDM, Hullenbergweg 83-85, 1101CL, Netherlands.

Contract Research Organization

MDS Pharma Services Italy, Centro Direzionale Milanofiori, Strada 4, Palazzo A/4, 20090 Assago (Milano), Italy. Phone number: +39 02 57514768; Fax number: +39 02 57790671.

4. INTRODUCTION

Influenza is characterized by the occurrence of frequent, unpredictable epidemics, and much less frequent, worldwide pandemics. Epidemics arise because different strains of influenza are constantly generated through antigenic drift and individuals are less or not at all protected in some years. A pandemic is responsible for higher morbidity and mortality than an epidemic because it affects a larger proportion of the population. The burden of epidemics, however, is cumulatively greater than that of pandemics. A worldwide pandemic is caused by the spread of a new influenza subtype arising from antigenic shift. When such a subtype enters the population, there is no natural immunity and the new virus can easily infect exposed individuals. In the 1957 pandemic ("Asian flu"), it took 5 months for the virus to travel from China to every continent and around 9 months for the pandemic to run its course. Although the great majority of deaths in current influenza epidemics occur among the elderly, a large proportion of influenza-related deaths in the 1918-1919 (H1N1), 1957-1958 (H2N2) and 1968-1969 (H3N2) pandemics were among those under 65 years of age. Over the first decade following each pandemic, this younger group accounted for decrementally smaller proportions of deaths. This pattern suggests that younger persons who survive infection with a new influenza A virus subtype develop long-term protection against fatal outcomes during subsequent epidemics of the same virus. Simonsen and colleagues predicted that in the next pandemic about half the associated deaths might occur initially among those less than 65 years of age. Furthermore, since during the 1957-1958 and 1968-1969 pandemics around two-thirds of deaths in the under 65's were in those aged 45-64 years, it would be prudent to consider targeting those aged 45-64 years during future pandemics and possibly during the following 10 years. International travel now facilitates the spread of virus around the world. Many novel influenza virus strains arise in China and South-east Asia, which are now common business and tourist destinations, again increasing the probability of novel strains being contracted and transported.

Once a pandemic begins, it will be too late to accomplish the many key activities required to minimize the impact. Therefore, planning and implementation of preparatory activities must start well in advance. The World Health Organization (WHO) has provided detailed guidance on the content of such plans. The 56th World Health Assembly adopted a specific resolution on May 28, 2003 to ensure that all WHO Member States give priority to influenza pandemic preparedness planning. This includes paying particular attention to the need to ensure adequate supplies of pandemic vaccine. Vaccines form the main prophylactic measure against pandemic influenza and in Europe play an important role in national and EU pandemic preparedness plans. Indeed, in the event of a pandemic, a specific monovalent vaccine against the emerging strain will have to be developed rapidly, then registered and produced in very large quantities. Speed in such vaccine development is vital and Regulatory Authorities produced Guidelines to provide the basis for a fast track licensing procedure for pandemic vaccines. In the EU, according to the EMEA, this procedure involves the submission and approval of a core pandemic dossier during the inter-pandemic period, followed by a fast track approval of the pandemic

vaccine, based on the submission of pandemic variation. Information in the core pandemic dossier should support a vaccination strategy that is likely to be used for a pandemic vaccine. To achieve this, a "mock-up" vaccine should be produced, ideally in the same way as the intended pandemic vaccine and have the same antigen content and adjuvant system (if used). The variation in case of a real pandemic can be submitted with a different antigen type.

Preliminary findings have identified the H2, H5, H6, H7 and H9 subtypes of influenza A virus as those most likely to be transmitted to humans and therefore presenting a potential pandemic threat. When reverse genetics approach is not applied, candidate vaccines most valuable for vaccine production are considered to be A/H7N1, A/H7N7, A/H7N3 and A/H9N2 G9 lineage. Recent A/H5N1 avian influenza cases in Asia have led to the use of reverse genetics in the preparation of an H5 pandemic strain. From 2003 until now, more than 115 human cases of H5N1 infection have been ascertained worldwide and 60 people died (more than 52% mortality rate). Virus left the original geographical region and appears today in mainland China, Philippines, Siberia and Europe. Tests conducted by the World Organisation for Animal Health (OIE) have confirmed the presence of highly pathogenic H5N1 avian influenza in samples taken from domestic birds in Turkey. In Romania, investigations of recent poultry deaths have, to date, identified the H5 subtype of avian influenza virus.

Previous clinical experience suggests that one dose of a pandemic vaccine, even in an adjuvanted formulation, is unlikely to induce a satisfactory immune response in unprimed, naïve individuals. Therefore the most likely scenario is that two doses of a pandemic vaccine will be necessary. Actually, a recent clinical trial has shown that for unadjuvanted vaccines 2 immunizations with 90 µg of strain-specific hemagglutinin (HA) are necessary to achieve protective levels of antibodies (M. Enserink "Pandemic Vaccine appears to protect only at high doses", Science, 309, 996, 12 August 2005), which means 12 times the normal 15 µg/dose required for the inter-pandemic seasonal influenza immunization. At the same time, use of an adjuvant allows reducing the quantity of antigen per dose and would potentially lead to increased vaccine production capacities. This is the only current practical solution to address the manufacturing capacity and to effectively respond to the global need for vaccination against a pandemic influenza.

To address the need for a pandemic vaccine, Novartis (formerly Chiron) is committed to developing, registering, producing and supplying influenza pandemic vaccines. Novartis Vaccines and Diagnostics S.r.l. (formerly Chiron S.r.l.) in Siena, Italy, is producing a A/H5N1 pandemic, surface antigen, inactivated influenza vaccine, with MF59C.1 (MF59) adjuvant, to be tested in clinical trials. Chiron (now Novartis) started dealing with H5N1 in 1997 and performed the first Phase I clinical trial against a pandemic influenza virus in 1999, using its MF59 adjuvanted, egg-derived, influenza pandemic vaccine formulation with a H5N3 strain. The pandemic FLUAD-like vaccine is a monovalent, surface antigen, inactivated influenza vaccine, adjuvanted with MF59, having as Drug Substance surface antigens from a virus strain candidate for a potential pandemic situation. The FLUAD-

like vaccine contains the same adjuvant and is manufactured with the same process used for FLUAD, a Novartis (formerly Chiron) inter-pandemic, trivalent influenza vaccine. FLUAD is licensed and marketed in many European countries and also outside Europe. The MF59 adjuvant contained in FLUAD is an oil-in-water emulsion, composed mainly of squalene, that is an intermediate metabolite in the synthesis of cholesterol. To date Chiron (now Novartis) has performed two Phase I clinical trials on the FLUAD-like influenza vaccine with two pandemic strains (H5N3 and H9N2). In both studies it was shown that two doses containing 7.5 µg of antigen are enough to reach protective levels of antibodies if the MF59 adjuvant is used. Anyway, the clinical picture is currently referred to two small size Phase I clinical trials and there is therefore the need to confirm the previous data with a statistically powered Phase II clinical trial.

The present study aims to evaluate safety and immunogenicity of two doses, administered three weeks apart, and a 6-months booster dose of two FLUAD-like influenza vaccines containing 7.5 µg or 15 µg of H5N1 influenza antigen, in non-elderly adult and elderly subjects.

A comprehensive review of influenza A/H5N1 pandemic subunit vaccine with MF59C.1 adjuvant is contained in the Investigator’s Brochure supplied by Chiron (now Novartis) Vaccines; this document should be reviewed prior to initiating the study.

5. STUDY OBJECTIVES

5.1 Immunogenicity Objectives

Immunogenicity Objectives

Primary

To assess immunogenicity of two 0.5 mL intramuscular (IM) injections of two FLUAD-like influenza vaccines containing either 7.5 µg or 15 µg of H5N1 influenza antigen, as measured by hemagglutination inhibition (HI) test in compliance with the requirements of the current European Union recommendations (CPMP/BWP/214/96).

Secondary

To compare immunogenicity of two 0.5 mL IM injections of two FLUAD-like influenza vaccines containing either 7.5 µg or 15 µg of H5N1 influenza antigen in terms of post-immunization geometric mean titers (GMTs) as measured by HI test.

To evaluate immunogenicity of one and three 0.5 mL IM injection of two FLUAD-like influenza vaccines containing either 7.5 µg or 15 µg of H5N1 influenza antigen, as measured by HI test.

To evaluate immunogenicity after one, two and three 0.5 mL IM injections of two FLUAD-like influenza vaccines containing either 7.5 µg or 15 µg of H5N1 influenza antigen, as measured by microneutralization (MN) test and possibly Single Radial Hemolysis (SRH) test in a subset of subjects selected in a 1:1 ratio with respect to the study vaccines and to the age group.

5.2 Safety Objectives

To evaluate the safety of the administration of three 0.5 mL IM injections of two FLUAD-like influenza vaccines containing either 7.5 µg or 15 µg of H5N1 influenza antigen.

6. OVERALL STUDY DESIGN

6.1 Overall Study Design and Plan – Description

6.1.1 Overall Study Design

460 evaluable subjects are necessary in this phase II, randomized, controlled, observer-blind, multi-center study. All subjects enrolled will be randomly assigned in a 1:1 ratio to one of the following vaccine groups:

Group A – two 0.5 mL injections, three weeks apart, of FLUAD-like influenza vaccine containing 7.5 µg of H5N1 influenza antigen, plus 0.5 mL booster injection at day 202 in, at least, the first half of the subjects enrolled in this group at each study site.

Group B – two 0.5 mL injections, three weeks apart, of FLUAD-like influenza vaccine containing 15 µg of H5N1 influenza antigen, plus 0.5 mL booster injection at day 202 in, at least, the first half of the subjects enrolled in this group at each study site.

Subjects will be observed for 30 minutes after each injection for any immediate reactions. All subjects will be instructed to complete a diary card to record local (i.e., ecchymosis, erythema, induration, swelling and pain at injection site) and systemic reactions (i.e., chills, malaise, myalgia, arthralgia, nausea, headache, sweating, fatigue and potential indicators of oculo-respiratory syndrome) and axillary temperature starting on the day of vaccination (after 6 hours) and for each of the 6 days following each immunization.

All adverse events will be collected from the day of vaccination and for 3 weeks following each immunization. All adverse events necessitating a physician's visit or consultation and/or leading to premature study discontinuation and all serious adverse events will be collected throughout the entire trial and data will be reconciled at study termination visit.

Serum samples for immunogenicity assays will be collected at day 1 (pre-immunization), at day 22, at day 43, at day 202 in all subjects and also at day 223 and 382 in the booster subset. Immunogenicity will be evaluated by HI test in all subjects, and additionally by MN and possibly SRH in a subset of at least 220 subjects for each test.
All subjects enrolled in this study will be informed that they may be invited to participate to an extension study approximately 12 months after the completion of this vaccination series.

See also section 6.5.5 for further details.

6.1.2 Planned Duration of Study

Expected subject enrollment interval: 8 weeks (approximately)

Duration of individual subject's participation: 29 weeks (approximately); 55 weeks (approximately) for the 6 months booster subset of subjects

Total duration of study: 37 weeks (approximately); 63 weeks (approximately) for the 6 months booster subset of subjects.

6.1.3 Premature Discontinuation of the Study

The sponsor, or the investigator (following consultation with the sponsor) has the right to discontinue this study at any time. If the clinical study is prematurely terminated, the investigator is to promptly inform the study subjects and should assure appropriate therapy and follow-up for the subjects. If the study is prematurely discontinued, all procedures and requirements pertaining to the archiving of the documents will be observed. All other study materials (completed, partially completed and blank CRFs, study medication/vaccines etc.) will be returned to the sponsor.

6.1.3.1 Stopping/Pausing Rules

There are no predetermined stopping rules other than those described in section 6.1.3.

6.2 Discussion of Overall Study Design

This study utilizes several standard features of clinical study design intended to reduce bias, including the random assignment of subjects to treatment groups and the use of an observer-blind design. Because study vaccines have slightly different appearance in the label, vaccinations and safety assessments will be each performed by different study personnel to ensure blinding. The subjects, their parents/legal guardians and the investigators will be blinded to the study vaccine given.

The design features were selected because of their well-recognized acceptance as scientifically appropriate.

6.3 Selection of Study Population

6.3.1 Inclusion Criteria

Subjects eligible for enrollment into this study are male and female adult volunteers who are:

1. 18 years of age or older, mentally competent, willing and able to give written informed consent prior to study entry;

2. able to comply with all the study requirements

3. in general good health as determined by:

- medical history
- physical examination
- clinical judgment of the investigator

Informed consent must be obtained for all the subjects before enrollment into the study.

6.3.2 Exclusion Criteria

Individuals are not to be enrolled into the study if:

1. they have any serious disease such as:
   - cancer (except for benign or localized skin cancer and non metastatic prostate cancer not presently treated with chemotherapy)
   - autoimmune disease (including rheumatoid arthritis)
   - advanced arteriosclerotic disease or complicated diabetes mellitus
   - chronic obstructive pulmonary disease (COPD) that requires oxygen therapy
   - acute or progressive hepatic disease
   - acute or progressive renal disease
   - congestive heart failure

2. they are hypersensitive to eggs, chicken protein, chicken feathers, influenza viral protein, neomycin or polymyxin or any other component of the vaccine;

3. they have a history of neurological symptoms or signs, or anaphylactic shock following administration of any vaccine

4. they have a known or suspected (or have a high risk of developing) impairment/ alteration of immune function (excluding that normally associated with advanced age) resulting, for example, from:
   - receipt of immunosuppressive therapy (any parenteral or oral cortico steroid or cancer chemotherapy/radiotherapy) within the past 60 days and for the full length of the study
   - receipt of immunostimulants
   - receipt of parenteral immunoglobulin preparation, blood products and/or plasma derivates within the past 3 months
   - suspected or known HIV infection or HIV-related disease

5. women who are pregnant, or women able to bear children but not willing to practice acceptable contraception for the duration of the trial;

6. within the past 4 weeks they have received:
   - another vaccine
   - any investigational agent

7. within the past 7 days, they have experienced:
   - any acute disease
   - infections requiring systemic antibiotic or antiviral therapy (chronic antibiotic therapy for urinary tract prophylaxis is acceptable)

8. within the past 3 days, they have experienced:
   - fever (i.e., axillary temperature ≥ 38°C);

9. they are taking part in another clinical study;

10. they have surgery planned during the study period

11. they have any condition which, in the opinion of the investigator, might interfere with the evaluation of the study objective.

6.3.3 Removal of Subjects from Therapy or Assessment

The subject, or where applicable, the subject's legally acceptable representative(s) can withdraw consent for participation in the study at any time without prejudice. The investigator can withdraw a subject if, in his or her clinical judgment, it is in the best interest of the subject or if the subject cannot comply with the protocol.

In addition, a subject may not be eligible for subsequent immunization or may be discontinued from the study following occurrence of:

- afebrile convulsions and neurological disturbances after vaccination
- hypersensitivity to the investigational vaccine
- other suspected side effects that could compromise the subject’s well being.

Any subject who, despite the requirement for adequate contraception, becomes pregnant during the trial will not receive further immunization. The site should maintain contact with the pregnant subject, complete a "Pregnancy Report" CRF as soon as possible, and obtain pregnancy outcome information for "Pregnancy Follow-up" CRF.

The subject will be clinically monitored after withdrawal, the cause of which will be recorded in detail on the “Study Termination” CRF, “Adverse Events” and/or “Concomitant Medications” CRF and, where appropriate, on the subject’s medical records. Where the withdrawal of a subject resulted from an adverse event, this will be documented in accordance with the procedures in section 6.5.4.5.

Whenever possible, the tests and evaluations listed for the termination visit will be carried out.

Withdrawn subjects will not be replaced.

6.4 Vaccines

6.4.1 Vaccines Administered

Subjects enrolled will be randomly assigned in a 1:1 ratio to one of the following vaccine groups to receive:

Group A (Vaccine A) – two 0.5 mL IM injections, three weeks apart, of FLUAD-like influenza vaccine containing 7.5 µg of H5N1 influenza antigen, plus a 0.5 mL IM booster injection at day 202 in, at least, the first half of the subjects enrolled in this group at each study site;

Group B (Vaccine B) – two 0.5 mL IM injections, three weeks apart, of FLUAD-like influenza vaccine containing 15 µg of H5N1 influenza antigen, plus a 0.5 mL IM booster injection at day 202 in, at least, the first half of the subjects enrolled in this group at each study site.

Both vaccines will be given in the deltoid region of the (preferably) non-dominant arm.

6.4.2 Identity of Investigational Vaccine

6.4.2.1 Vaccine Composition

Vaccine A: FLUAD-like influenza vaccine containing 7.5 µg of the A/Vietnam/1194/2004-like (H5N1) influenza antigen**.**

**Name of ingredients**	**Quantity per dose**
**Active substances:**	
A/Vietnam/1194/2004-like (H5N1)	≥ 7.5 µg
**Excipients:**	
sodium chloride	4.00 mg
potassium chloride	0.10 mg
potassium dihydrogen phosphate	0.10 mg
disodium phosphate dihydrate	0.66 mg

calcium chloride dihydrate	0.06 mg
magnesium chloride hexahydrate	0.05 mg
water for injections	up to 0.50 mL
**Adjuvants:**	
Squalene	9.75 mg
polysorbate 80	1.175 mg
sorbitan trioleate	1.175 mg
sodium citrate	0.66 mg
citric acid	0.04 mg

Vaccine B: FLUAD-like influenza vaccine containing 15 µg of the A/Vietnam/1194/2004-like (H5N1) influenza antigen**.**

**Name of ingredients**	**Quantity per dose**
**Active substances:**	
A/Vietnam/1194/2004-like (H5N1)	≥ 15 µg
**Excipients:**	
sodium chloride	4.00 mg
potassium chloride	0.10 mg
potassium dihydrogen phosphate	0.10 mg
disodium phosphate dihydrate	0.66 mg
calcium chloride dihydrate	0.06 mg
magnesium chloride hexahydrate	0.05 mg
water for injections	up to 0.50 mL
**Adjuvants:**	

Squalene	9.75 mg
polysorbate 80	1.175 mg
sorbitan trioleate	1.175 mg
sodium citrate	0.66 mg
citric acid	0.04 mg

6.4.2.2 Vaccine Labeling, Storage and Packaging

All vaccine supplies must be stored between +2/+8°C. Vaccines that have been stored differently from the sponsor's recommendations **must not** be used unless the sponsor provides written authorization for use. In the event that the use cannot be authorized, vaccine supply must be replaced with fresh stock supplied by the sponsor.

The sponsor will supply the investigational vaccines. The investigator (or pharmacist) will make an inventory and acknowledge receipt of all shipments of study vaccines.

The study vaccines will be supplied and packaged as a single dose per carton in a pre-filled syringe.

The label attached on the carton will contain the following information:

<u>Vaccine A</u>

V87P1 Vaccine A Site No. Investigator name
FLUAD-like pandemic influenza vaccine H5N1 Lot: 050201A Expiry date: 10/2006
containing 7.5 mg of H5N1 influenza
antigen
Influenza Vaccine Surface Antigen, Inactivated, Adjuvanted with MF59C.1
0.5 ml of suspension for injection in an emulsion in pre-filled syringe containing 7.5µg HA of H5N1 influenza antigen
0.5 ml/dose
i.m. injection only
Warning: do not inject intravascularly or subcutanously.
Store at +2/+8 °C. Protect from light. Do not freeze.
Allow the vaccine to reach the room temperature and gently shake before use.
Keep the drug out of reach and sight of children.
Caution: For clinical trial use only CHIRON S.r.l. – Siena – Italy

Vaccine B

V87P1 Vaccine B Site No. Investigator name
FLUAD-like pandemic influenza vaccine H5N1 Lot: W52P04H1 Expiry date: 01/2007
containing 15 mg of H5N1 influenza
antigen
Influenza Vaccine Surface Antigen, Inactivated, Adjuvanted with MF59C.1
0.5 ml of suspension for injection in an emulsion in pre-filled syringe containing 7.5µg HA of H5N1 influenza antigen
0.5 ml/dose
i.m. injection only
Warning: do not inject intravascularly or subcutanously.
Store at +2/+8 °C. Protect from light. Do not freeze.
Allow the vaccine to reach the room temperature and gently shake before use.
Keep the drug out of reach and sight of children.
Caution: For clinical trial use only CHIRON S.r.l. – Siena – Italy

The label attached on the pre-filled syringe will contain the following information:

Vaccine A

V87P1 influenza vaccine
H5N1 Pandemic FLUAD-like –
1 dose (7.5 µg HA in 0.5 ml)
i.m. injection only (in the deltoid muscle)
Gently shake before use
Lot: 050201A Expiry date: 10/2006
Store at +2/+8 °C
Caution: For clinical trial use only
CHIRON S.r.l. – Siena – Italy

Detachable part:
V87P1-Vaccine A
Subject Number
Initials:
Date

Vaccine B

V87P1 influenza vaccine
H5N1 Pandemic FLUAD-like –
1 dose (15 µg HA in 0.5 ml)
i.m. injection only (in the deltoid muscle)
Gently shake before use

Lot: W52P04H1 Expiry date: 01/2007
Store at +2/+8 °C
Caution: For clinical trial use only
CHIRON S.r.l. – Siena – Italy

Detachable part:
V87P1-Vaccine B
Subject Number
Initials:
Date

Note: The expiry date of the current vaccines lot provided may precede the visit window of the 6 month booster.
Based on new stability data the sponsor will either extend the expiry dates or provide the sites with a fresh vaccine lot according to Novartis SOP's.

6.4.2.3 Vaccine Administration

The investigator will be responsible for the administration of the vaccine to subjects enrolled into the study according to the procedures stipulated in this study protocol. All vaccines will be administered only by unblinded personnel who are qualified to perform that function under applicable local laws and regulations for the specific study site.

The vaccine must be **well shaken** and visually inspected before use. The vaccination site should be disinfected with a skin disinfectant (e.g. 70% alcohol). Before vaccination, the skin must be dry. **DO NOT inject intravascularly**.

PRECAUTIONS TO BE OBSERVED IN ADMINISTERING STUDY VACCINE:

Study vaccines should not be administered to individuals with known hypersensitivity to any component of the vaccines.

Any axillary temperature ≥ 38°C or serious active infection is reason for delaying vaccination. Immunization should also be delayed according to instructions in section 6.3.3.

Standard immunization practices should be observed and care should be taken to administer the IM injection. As with all injectable vaccines, appropriate medical treatment and supervision should be readily available in case of rare anaphylactic reactions following administration of the study vaccine. Epinephrine 1:1000 and diphenhydramine should be available in case of any anaphylactic reactions. Care must be taken to ensure the vaccine is not injected into a blood vessel.

6.4.3 Method of Assigning Subjects to Vaccination Groups

Subjects who meet the study admission criteria will be enrolled into the study and will be assigned a 5-digit subject number. The first two digits identify the study site. The next three digits identify the subject within the site and will be assigned sequentially, with 001 corresponding to the first subject enrolled in the 18-60 years age group (i.e., ≥ 18 and < 61) and with 301 corresponding to the first subject enrolled in the 61 years and over age group (i.e., ≥ 61) at any particular site.

Two randomization lists, one for each age group, will be provided to each Investigator by Novartis Vaccines and will be used only by the unblinded study personnel to assign the subjects to the vaccination groups.

6.4.4 Selection of Doses in the Study

The dosage and the schedule used in this study are based on data from preceding studies.

6.4.5 Blinding

The trial is designed as an observer-blind study. During the study, designated nurses or physicians will be responsible for administering the study vaccines to the subjects, and will be instructed not to reveal the identity of the study vaccines neither to the subject nor to the investigative site personnel (investigator, study nurse) involved in the monitoring or conduct of the trial, except in an emergency. These designated individuals will have no contact with the subjects after the administration of the study vaccine. If the study vaccine code is supplied to the investigator in the event of an emergency, the Study / Site Monitor must be notified immediately by the investigator. The date and time, along with the reason for the unblinding, should be noted. Study vaccine codes will not be freely available to the investigator or personnel monitoring the trial until after the completion of the trial and final data review.

6.4.6 Prior and Concomitant Therapy

During this trial medication prescribed to the subject prior to the start of the study will not be collected. All prescription medication, including non-study vaccines, being taken by the subjects on entry to the study and all prescription medication given in addition to the study vaccine during this clinical trial are to be regarded as concomitant medication and must be documented on the “Concomitant Medications” CRF.

The following concomitant treatments are discouraged and, if used, might lead to a major protocol violation according to the medical judgment of the Novartis physician (see exclusion criteria):

- Systemic steroids

- Other immunosuppressive agents
- Blood or plasma derivates, including immunoglobulin
- Non-study vaccines (with the exception of post-exposure vaccinations in a medical emergency, e.g., hepatitis, rabies, tetanus). In consideration of the overlapping Northern Hemisphere influenza vaccination campaign and the visit window of the 6 months booster, the use of flu vaccines is also discouraged for the 3 weeks following the booster.

The use of inter-pandemic influenza vaccines

6.4.7 Vaccination Compliance

The investigator will be responsible for adequate and accurate accounting of vaccine usage. The investigator or designee will administer the study vaccines only to individuals included in this study following the procedures set out in this study protocol. The date, dosage, and time of the vaccinations will be recorded. The investigator will track vaccines received, used and wasted and will retain all unused or expired products until the sponsor is satisfied that the vaccine accountability records are correct. Thereafter, all unused vaccines are to be returned to the sponsor or destroyed at the investigational site according to the relevant Novartis SOP. An overall summary of vaccines supplied, received, wasted, used and returned will be prepared at the conclusion of the study.

6.4.8 Adherence to Randomization List

An unblinded, appropriately qualified person designated by the investigator will prepare/administer the vaccine as indicated in the randomization lists for the individual subject. The investigator as well as the Study / Site Monitor will be blinded during the whole conduct of the study.

Adherence to randomization lists will be verified by Novartis Clinical Quality Assurance (CQA) by checking the randomization lists against the vaccination records. A copy of the vaccine accountability logs and administration logs is to be sent to CQA, Novartis Vaccines and Diagnostics S.r.l., Via Fiorentina, 1, 53100 Siena, Italy, Fax +39 0577 278507, weekly during the enrollment period of this study.

6.5 Immunogenicity and Safety Variables

6.5.1 Immunogenicity and Safety Measurements Assessed

The Time and Events Table in the Synopsis and section 6.5.5 describes the required evaluations.

6.5.2 Appropriateness of Measurements

The measures of immunogenicity used in this study are standard, i.e., widely used and generally recognized as reliable, accurate, and relevant (able to describe the quality and extent of the immune response).

The measures of safety used in this study are routine clinical procedures. They include a close vigilance for, and stringent reporting of, selected local and systemic reactions routinely monitored in vaccine clinical trials as indicators of reactogenicity.

6.5.3 Immunogenicity Variables

The measures of immunogenicity, collected for all evaluable subjects by using HI test and, in a subset of subjects, by using MN and possibly also SRH test, are:

- the GMTs (GMAs) at day 1, at day 22, at day 43, at day 202, at day 223 and at day 382 as determined by HI, SRH and MN.
- the day 22/day 1, the day 43/day 22, the day 43/day 1, the day 202/day 43, the day 202/day 1 and the day 223/day 202 and the day 223/day 1, the day 382/day 223, the day 382/day 202 and the day 382/day 1 geometric mean titer ratios (GMRs) as determined by HI, SRH and MN.
- the percentage of subjects achieving seroconversion[1] or significant increase in antibody titer[2] at day 22, at day 43, at day 202, at day 223 and at day 382, as determined by HI and SRH.
- the percentage of subjects with at least a four-fold rise in titer at days 22, at day 43, at day 202, at day 223 and at day 382, as determined by HI, SRH and MN.
- the percentage of subjects achieving a titer ≥ 40 (an SRH area ≥ 25mm^2) at day 1, at day 22, at day 43, at day 202, at day 223 and at day 382, as determined by HI, SRH and MN.

[1] Seroconversion is defined as negative pre-vaccination serum / post-vaccination titer ≥ 40 (area ≥ 25 mm^2).
[2] Significant increase in antibody titer is defined as at least a fourfold increase from non-negative pre-vaccination serum (a 50% increase in area).

6.5.4 Safety Variables

A brief medical history will be obtained and physical examination performed for each subject entered into the study.

Local and systemic reactions and other adverse events will be collected throughout the study, as detailed in sections 6.1.1 and 6.5.5.

6.5.4.1 Adverse Events

An adverse event (AE) is defined as any untoward medical occurrence in a patient or clinical investigation subject administered a pharmaceutical product at any dose that does not necessarily have to have a causal relationship with this treatment. An AE can, therefore be any unfavorable and unintended sign (including an abnormal laboratory finding, for example), symptom, or disease temporally associated with the use of an investigational product, whether or not considered related to the investigational product. This definition includes intercurrent illnesses or injuries and exacerbation of pre-existing conditions.

An *unexpected adverse event* is one that is not listed in the current Summary of Product Characteristics or the Investigator's Brochure or an event that is by nature more specific or more severe than a listed event.

All AEs will be monitored until resolution or, if the AE becomes chronic, a cause identified. If an AE is unresolved at the conclusion of the study, a clinical assessment will be made by the investigator and Medical Monitor whether continued follow-up of the AE is warranted.

The severity of events reported on the "Adverse Events" CRF will be determined by the investigator as:

Mild:	transient with no limitation in normal daily activity.
Moderate:	some limitation in normal daily activity.
Severe:	unable to perform normal daily activity.

The relationship of the study treatment to an AE will be determined by the investigator based on the following definitions:

<u>1. Not Related</u>

The AE is not related if exposure to the investigational vaccine has not occurred, **or** the occurrence of the AE is not reasonably related in time, **or** the AE is considered unlikely to be related to use of the investigational vaccine, i.e. there are no facts (evidence) or arguments to suggest a causal relationship.

<u>2. Possibly Related</u>

The administration of the investigational vaccine and AE are considered reasonably related in time **and** the AE could be explained by causes other than exposure to the investigational vaccine.

<u>3. Probably Related</u>

Exposure to the investigational vaccine and AE are reasonably related in time **and** the investigational vaccine is more likely than other causes to be responsible for the AE, **or** is the most likely cause of the AE.

The relationship of the study treatment to an AE will be determined by the investigator.

6.5.4.2 Serious Adverse Events

A serious adverse event (SAE) is defined as any untoward medical occurrence that at any dose:

- Results in death
- Is life-threatening (i.e., the subject was, in the opinion of the investigator, at immediate risk of death from the event as it occurred); it does not refer to an event which hypothetically might have caused death if it were more severe.
- Requires or prolongs inpatient hospitalization
- Results in persistent or significant disability/incapacity (i.e., the event causes a substantial disruption of a person's ability to conduct normal life functions)
- Results in a congenital anomaly/birth defect
- Requires intervention to prevent permanent impairment or damage
- Is an important and significant medical event that may not be immediately life threatening or resulting in death or hospitalization but, based upon appropriate medical judgment, may jeopardize the patient/subject or may require intervention to prevent one of the other outcomes listed above.

Of note: a "possible vaccine failure" should be reported as a serious AE only if it resulted in an infectious disease which should have been prevented by the vaccine implied.

Adverse events which do not fall into these categories are defined as **non-serious**.

It should be noted that a severe adverse event need not be serious in nature and that a serious adverse event need not, by definition, be severe.

In addition, a pre-existing event or condition that results in hospitalization should be recorded on the "Medical History" CRF. The hospitalization would not result in the event or condition being reported as an on study SAE unless, in the view of the investigator, hospitalization was prolonged as a result of participation in the clinical trial or was necessary due to a worsening of the pre-existing condition. This is because the onset of the event (the reason for the procedure) occurred before the subject was entered in the

trial. Hospitalization for cosmetics, non-emergency prophylaxis or abortion does not result in an SAE report unless, in the view of the investigator, hospitalization for these procedures was prolonged as a result of participation in the clinical trial.

6.5.4.3 Local and Systemic Reactions

The occurrence of selected indicators of reactogenicity (listed below), which by definition, can only occur up to 6 days post-vaccination, will be recorded on the "Local and Systemic Reactions" rather than the "Adverse Events" CRF.

Local Reactions:

Ecchymosis, erythema, induration, swelling and pain at injection site.

Systemic Reactions:

Chills, malaise, myalgia, arthralgia, nausea, headache, sweating, fatigue and potential indicators of oculo-respiratory syndrome.

Other indicators of Reactogenicity:

Stayed at home due to reactions, axillary temperature and use of analgesic/antipyretic medication.

6.5.4.4 Clinical Laboratory Tests

Not applicable.

6.5.4.5 Documentation/Monitoring/Reporting of Safety Variables

All study subjects will be observed for at least 30 minutes after each vaccination for evidence of immediate reactions in general and in particular for symptoms of allergic phenomena (such as rashes, itching, or other allergic manifestations). Each subject, or where applicable, the subject's legally acceptable representative(s) will be instructed to complete a diary card for 7 days (including the day of vaccination) following each administration, to describe local reactions and other selected indicators of reactogenicity. If a local and systemic reaction continues beyond 6 days after a vaccination, it will also be recorded on the "Adverse Events" CRF. If the subject recovers on the last day, then this fact will be recorded on the "Comments" CRF.

All AEs must be reported and documented. The period of observation for adverse events extends from the time the subject gives informed consent until he or she undergoes the final study examination. This may include a period before and after an active treatment of an investigational product (study vaccine) or other medication.

All adverse events, regardless of severity, will be monitored by the investigator until resolution. All subjects experiencing adverse events - whether considered associated with the use of the study vaccine or not - must be monitored until symptoms subside and any abnormal laboratory values have returned to baseline, or until there is a satisfactory explanation for the changes observed, or until death, in which case a full pathologist's report should be supplied, if possible. All findings must be reported on an “Adverse Events” CRF and on the “Vaccine Serious Adverse Event” form, if necessary, which is part of the investigator’s study file. All findings in subjects experiencing adverse events must be reported also in the subject's medical records.

In addition, any event resulting in a subject’s withdrawal from subsequent vaccinations or from follow-up should be reported according to the protocol instructions. All SAEs which occur during the course of the trial, whether considered to be associated with the study vaccination or not, have to be reported **within 24 hours** or at the latest on the following working day by telephone or fax to either of the following:

Medical Monitor: Angelika Banzhoff, MD
P.O. Box 1630
35006 Marburg, Germany
Phone number: +49 6421 392681
Fax: +49 6421 394667

Study Monitor: Massimo Bianchini, PhD
Phone number: +39 0577 243428
Fax: +39 0577 243551

For trial related **emergencies out of office hours** please contact: +49 (0)151 12641663.

As far as possible, all points raised on the “Vaccine Serious Adverse Event” form need to be addressed and faxed immediately to the Medical Monitor or Study Monitor. Afterwards the original form must be sent by post to the Medical Monitor/Study Monitor. The event must also be documented on the “Adverse Events” CRF. After receipt of the initial report, the Medical Monitor/Study Monitor will review the information and contact the investigator if it is necessary to obtain further information for assessment of the event. Any medication or other therapeutic measures used to treat the event will be recorded on the appropriate CRF(s) in addition to the outcome of the AE. Any serious or unexpected AE must be reported to the EC in a timely manner. Adequate documentation will be provided to the sponsor showing that the EC has been properly notified.

If required, a follow-up report including all new information obtained on the serious adverse event must be prepared and sent to the Study Monitor, or it will be collected by a representative of the sponsor. The report should be marked "Follow-up report".

The investigator will submit, on request, copies of all these reports to the EC and other relevant authorities.

Post-Study Events

Any AE occurring at any time outside the observation period or after the end of the study and considered to be caused by the study vaccine - and therefore a possible adverse drug reaction - must be reported.

6.5.5 Study Procedures and Flowchart

Informed consent must be obtained from the subject, or where applicable, the subject's legally acceptable representative(s) prior to the performance of any trial specific tests or evaluations, i.e., any unusual or non-routine procedures that involve risk, however trivial, to the subject.

TIME AND EVENTS TABLE

**Study Visit**	**1**	**2**	**3**	**4**	**5**	**6**
**Study Day (window)**	**1**	**22 (21-25)**	**43 (42-46)**	**202 (188-216)**	**223 (222-226)**	**382 (378-386)**
Obtain Informed Consent	X			X		
Medical History	X					
Physical Examination	X	X	X	X	X	X
Assess Incl./Excl. Criteria	X			X		
Obtain Blood Sample	X	X	X	X	X	X
Administer Study Vaccine	X	X		X		
Local and Systemic Reactions	X	X	X	X	X	
Dispense Diary Card*	X			X		
Assess Adverse Events	X	X	X	X***	X	X***
Concomitant Medication	X	X	X	X***	X	X
Diary Card Review**		X	X		X	
Study Termination				X		X

* The diary cards will be maintained by each subject from Visit 1 to Visit 3 and from Visit 4 to Visit 5.

** Review the Diary Cards at Visits 2 and 3 and at Visit 5 and record the data on the "Local and Systemic Reaction" CRFs. At the end of Visits 3 and 5 collect the Diary Cards for filing in the Investigator's Study File. Reconcile Adverse Events and Concomitant Medication on the CRF pages if applicable.

*** At Visit 4 and 6, only Serious Adverse Events and/or Adverse Events necessitating a physician's visit or consultation and/or leading to premature study discontinuation will be recorded as well as Concomitant Medication used for these events.

1) Visit 1, Day 1

a. Explain and obtain written informed consent from each subject. Informed consent must be obtained prior to performance of any study-specific tests or evaluations. [NOTE: "study-specific tests or evaluations" are defined as unusual or non routine procedures that involve risk, however trivial, to the subject]

b. Obtain and record significant medical history.

c. Perform brief physical examination, including measurement of heart rate, respiratory rate, and including check of the heart, lungs, and axillary lymph nodes, etc.

d. Review, with females of childbearing potential, their ability to become pregnant and commitment to practice appropriate birth control as determined by the investigator for the duration of the study. If sexually active, the subject must have been using one of the accepted birth control methods for at least two months prior to study entry. A urine pregnancy test will be performed on all female subjects of childbearing potential. Complete the pregnancy test form as appropriate (see exclusion criterion 5).

e. If the subject meets all inclusion and no exclusion criteria, assign a study subject number.

f. Collect pre-immunization blood sample (10 mL) for immunogenicity assays.

g. Measure axillary temperature and check the non-dominant arm chosen for injection immediately prior to immunization.

h. The designated study vaccine administrator will administer intramuscularly the study vaccine, according to the randomization list.

i. At the end of 30 minutes, take the subject's axillary temperature and examine the vaccine injection site for local reactions. Record these findings and any systemic reactions or AEs if applicable on the subject's source documents and appropriate CRF.

j. Instruct each subject in the evaluation and registration of local and systemic reactions, axillary temperature, adverse events and concomitant medications.

k. Give the subject a thermometer to measure axillary temperature at home and instruct the subject in its use.

l. Dispense the diary card for vaccination reactions to the subjects, and give instructions for its completion. The diary card must be returned to the site at Visit 2.

m. Schedule Visit 2 (Day 22; window 21-25).

2) Visit 2, Day 22 (window: -1/+3 days)

a. Obtain interim medical history (with reconciliation of subject's diary card) including local and systemic reactions, axillary temperature, adverse events and prescription medications.

b. Perform brief physical examination, including measurement of heart rate, respiratory rate, and including check of the heart, lungs, and axillary lymph nodes, etc. Record these findings on the subject's source documents and, if applicable, on the appropriate CRF.

c. Assess the subject for inclusion and exclusion criteria (including childbearing status and prosecution of accepted birth control methods) and for any contraindication to receive further vaccination.

If the subject continues to meet all inclusion and no exclusion criteria:

d. Collect pre-immunization blood sample (10 mL) for immunogenicity assays.

e. Measure axillary temperature and check the non-dominant arm chosen for injection immediately prior to immunization.

f. The designated study vaccine administrator will administer intramuscularly the study vaccine, according to the randomization list.

g. At the end of 30 minutes, take the subject's axillary temperature and examine the vaccine injection site for local reactions. Record these findings and any systemic reactions or AEs if applicable on the subject's source documents and appropriate CRF.

h. Instruct again each subject in the evaluation and registration of local and systemic reactions, axillary temperature, adverse events and concomitant medications.

i. Return the diary card for vaccination reactions to the subjects, and give instructions for its completion. The diary card must be returned to the site at Visit 3.

j. Schedule Visit 3 (21 days after visit 2; window 20-24 days after visit 2).

If the subject was not suitable to continue study participation, record study termination information on subject's source documents and, appropriate, CRF.

3) Visit 3, Day 43 (window: -1/+3 days)

a. Obtain interim medical history (with reconciliation of subject's diary card) including local and systemic reactions, axillary temperature, adverse events and prescription medications.
b. Perform brief physical examination, including measurement of heart rate, respiratory rate, and including check of the heart, lungs, and axillary lymph nodes, etc. Record these findings on the subject's source documents and, if applicable, on the appropriate CRF.
c. Collect blood sample (10 mL) for immunogenicity assays.
d. Instruct the subject in the registration of adverse events and concomitant medications.
e. Schedule Visit 4, Day 202 (window 188-216).

4) Visit 4, Day 202 (window: ± 14 days)

a. Ask for any SAE and/or AE necessitating a physician's visit or consultation that occurred form Visit 3 to Visit 4 and record them on the appropriate CRF. Review "Adverse Events" and "Concomitant Medication" CRFs, if appropriate. Record any pregnancies and follow up.
b. Perform brief physical examination, including measurement of heart rate, respiratory rate, axillary temperature, and including check of the heart, lungs, and axillary lymph nodes, etc. Record these findings on the subject's source documents and, if applicable, on the appropriate CRF.
c. Explain and obtain written informed consent from each subject. Informed consent must be obtained prior to performance of any study-specific tests or evaluations. [NOTE: "study-specific tests or evaluations" are defined as unusual or non routine procedures that involve risk, however trivial, to the subject].

If the subject has not given the consent neither to the 6 months blood draw nor to the booster and subsequent blood draw at day 223 and 382 (COHORT A):

d. Record study termination information on subject's source documents and, appropriate, CRF.

Inform the subjects that they may be invited to participate to an extension study approximately 12 months after the completion of this vaccination series.

If the subject has given the consent to the 6 months blood draw only (COHORT B):

d. Collect blood sample (10 mL) for immunogenicity assays.

e. Record study termination information on subject's source documents and, appropriate, CRF.

f. Inform the subjects that they may be invited to participate to an extension study approximately 12 months after the completion of this vaccination series.

If the subject has given the consent both to the 6 months blood draw and to the booster and subsequent blood draws at day 223 and 382 (COHORT C):

d. Collect blood sample (10 mL) for immunogenicity assays.

e. Review, with females of childbearing potential, their ability to become pregnant and commitment to practice appropriate birth control as determined by the investigator throughout study termination visit. A urine pregnancy test will be performed on all female subjects of childbearing potential. Complete the pregnancy test form as appropriate (see exclusion criterion 5).

f. If the subject continues to meet all inclusion and no exclusion criteria, measure axillary temperature and check the non-dominant arm chosen for injection immediately prior to immunization.

g. The designated study vaccine administrator will administer intramuscularly the study vaccine, according to the randomization list.

h. At the end of 30 minutes, take the subject's axillary temperature and examine the vaccine injection site for local reactions. Record these findings and any systemic reactions or AEs if applicable on the subject's source documents and appropriate CRF.

i. Instruct each subject in the evaluation and registration of local and systemic reactions, axillary temperature, adverse events and concomitant medications.

j. Dispense the diary card for vaccination reactions to the subjects, and give instructions for its completion. The diary card must be returned to the site at Visit 5.

k. Each subject will be invited to avoid taking any influenza vaccine during the following three weeks (i.e. until visit 5). To this extend the investigator will inform the subject that a Novartis marketed influenza vaccine Agrippal®S1 can be administered at the site free of charge at visit 5.

l. Schedule visit 5 (21 days after visit 4; window 20-24 days after visit 4).

5) Visit 5, Day 223 (window: -1/+3 days)

a. Obtain interim medical history (with reconciliation of subject's diary card) including local and systemic reactions, axillary temperature, adverse events and prescription medications. Review "Adverse Events" and "Concomitant Medication" CRFs, if appropriate. Record any pregnancies and follow up.

b. Perform brief physical examination, including measurement of heart rate, respiratory rate, and including check of the heart, lungs, and axillary lymph nodes, etc. Record these findings on the subject's source documents and, if applicable, on the appropriate CRF.

c. Collect blood sample (10 mL) for immunogenicity assays.

d. Schedule visit 6 (day 382; window 378-386)

6) Visit 6, Day 382 (window: ± 4 days)

a. Ask for any SAE and/or AE necessitating a physician's visit or consultation that occurred from Visit 5 to Visit 6 and record them on the appropriate CRF. Review "Adverse Events" and "Concomitant Medication" CRFs, if appropriate. Record any pregnancies and follow up.

b. Perform brief physical examination, including measurement of heart rate, respiratory rate, and including check of the heart, lungs, and axillary lymph nodes, etc. Record these findings on the subject's source documents and, if applicable, on the appropriate CRF.

c. Collect blood sample (10 mL) for immunogenicity assays.

d. Record study termination information on subject's source documents and, appropriate, CRF.

e. Inform the subjects that they may be invited to participate to an extension study approximately 12 months after the completion of this vaccination series.**6.5.5.1 Study Evaluations**

Blood samples taken in the following time windows will be evaluable:

Pre-immunization:	day 1
Post-immunization:	day 22 (window: 21 – 25, 20-24 days after visit1)
	day 43 (window: 42 – 46; 20-24 days after visit 2)
	day 202 (window 188-216; 166-194 days after visit 2)
	day 223 (window 222-226; 20-24 days after visit 4)
	day 382 (window 378-386; 176-184 days after visit 4)

6.5.5.1.1 Laboratory Assessments

No laboratory assessments will be made during this study.

6.5.5.1.2 Labeling and Storage of Serum Samples for Serology

Approximately 5 mL serum should be available for assays.

The blood will be centrifuged on the same day and the serum will be distributed in 2 aliquots for all subjects and in 3 aliquots for the first 28 subjects of each age group at each site, who will be also tested by MN and possibly SRH. The aliquots will be stored at a temperature of minus 18°C or below.

Should site 02 not reach the number of 28 subjects per age group, the missing aliquots for MN and possibly SRH test will be obtained at the MN laboratory. In particular, the laboratory will obtain the missing aliquots consecutively from site 03 samples, starting from the 29th subject of each age group.

Each serum tube will be labeled with an identifying bar code including at least subject number, subject initials, protocol number, visit number and aliquot identifier. Serum samples will be sent to the sponsor or will be collected by a representative of the sponsor.

For further information see Appendix B.

Complete instructions for labeling and storage of serum samples are included in the Study Reference Manual.

6.6 Study Monitoring, Auditing and Documentation

Investigators and/or their study staff will be trained at the latest during the initiation visit. During each monitoring visit source data verification will be performed by qualified staff representing the sponsor. A CRF collation supplied by the sponsor will be completed for each subject. The entries will be checked by trained delegates of the sponsor.

Monitoring and auditing procedures of the sponsor will be followed, in order to comply with GCP guidelines and to ensure validity of the study data.

The sponsor's CQA department will review the study documentation used for planning, conduct and monitoring of the study in order to ensure compliance with GCP and local regulations. This documentation includes as a minimum: the Study Protocol, the Case Report Forms, the Analysis Plan and the Subject Information and Consent Form.

6.6.1 Study Monitoring

The clinical study site will be monitored by regular site visits and telephone calls to the investigator by members of the Clinical Research department, the sponsor, or their agents following Novartis internal and/or CRO SOP. For this study, the expected average monitoring frequency is every 2 weeks by personal visit. The first monitoring visit to each investigational site will be performed not later than 1 days after first subject enrollment at the study site. By frequent communication, the site monitor will ensure that the study is conducted according to the protocol.

CRFs and all original data collected at the site should be available for review during monitoring visits. During these visits, the site monitor should review drug accountability records and document retention including the Investigator's Study File. Additionally, the site monitor should check that clinical study procedures are observed and discuss any problems with the investigator.

6.6.2 Source Data Verification and Audits

Inspection and examination of CRFs and source documents (all original recordings, laboratory reports, medical records) - giving due consideration to data protection and medical confidentiality - will be undertaken by representatives of the sponsor. All data not recorded directly on the CRFs as defined in section 6.10 of this study protocol must be verified by checking CRF entries against source documents in order to ensure that the data have been completely and accurately reported as required by the study protocol. Source data verification will be performed and recorded following Novartis (formerly Chiron) internal and/or CRO SOP. The subject or the subject's legally acceptable representative must also allow access to the subject's medical records. Each subject, or the subject's legally acceptable representative, will be informed of this prior to the start of the study.

During or after the clinical study, the regulatory authorities, the EC and/or representatives of the sponsor may request access to all source documents, CRFs and other study documentation for on-site audit or inspection.

6.7 Data Management

All CRF data (except for comments fields on the "Medical History" and "Comments" CRFs and the "Other, specify" item on the "Screen Log", if applicable), will be entered in duplicate into a Clintrial® database by BCDM, Novartis.

Data validation will be performed using the programming languages PL-SQL and SAS® version 9.1 or higher (SAS Institute, Cary, NC). Data quality assurance will be performed by doing a 100% check of all database updates resulting from the resolution of queries, and by estimating the database error rate with 95% upper confidence limit. The latter must be below the departmental accepted level of 0.5%. Analysis will be performed by BCDM using SAS® version 8.2 or higher according to a predefined Analysis Plan (AP), developed by the study Biostatistician.

All serology data analyzed by Clinical Serology, Novartis Vaccines, Marburg will be entered into the Seroad database by Clinical Serology, Novartis Vaccines, Marburg. All results will be checked in the laboratory for validity and completeness by three persons before access to the data is granted to BCDM, Novartis.

Electronic Data Transfer (EDT) is one method being used by Novartis for collecting data generated by an external laboratory. The full-service laboratory (i.e., central laboratory)

will send data as electronic files by a secured method (e.g., via diskette, CD, as an encrypted file attachment on electronic mail, or as a direct transfer into a specified VMS directory) to the BCDM department, Novartis. The data file is pre-processed and loaded by the BCDM Lab Manager into the study database. The laboratory will submit a RESULTS file containing the tests and the results as specified in the protocol. If the laboratory provides the service, it will also submit a Demography (DEMOG) file containing the subject's demographic information. If the file includes results of data blinded to Clinical, the source will provide a separate RESULTS file that will be loaded into a separate laboratory table.

6.7.1 Data Handling Procedures

Coding will be performed using the following dictionaries:

Adverse Events:	MedDRA
Concomitant illness:	ICD-9
Concomitant and intercurrent therapy:	WHO Drug Dictionary

6.7.2 Data Protection

Novartis respects the subjects' rights to privacy and will ensure the confidentiality of their medical information in accordance with all applicable laws and regulations.

The sponsor as Data Controller according to the European Directive on the protection of individuals with regard to the processing of personal data and on the free movement of such data [95/46/EC] confirms herewith compliance to Directive 95/46/EC in all stages of Data Management.

6.8 Changes in the Conduct of the Study or Planned Analysis

Planned changes in the conduct of the study will be described in protocol amendments; changes in the planned analysis will be described in the clinical study report.

An amendment is a written description of change(s) to or formal clarification of a study protocol.

The EC must be informed of all amendments and if necessary prior review and documented approval/favorable opinion must be sought for ethical aspects. Approval must also be obtained from the authorities, if necessary.

Such amendment will be agreed upon by the sponsor, the investigator, the EC, and authorities if necessary prior to implementation.

6.9 Statistical Methods and Determination of Sample Size

6.9.1 Statistical Plans

The statistical evaluation of the results will be performed by BCDM as predefined in the AP. The statistical tables and graphs will be generated using SAS® version 9.1 or higher.

Definition of populations to be analyzed:

(a) All-randomized population, Demography and subject listings.

- All subjects who have a record in the DEMOG panel

(b) Intention-to-treat (ITT) population, Immunogenicity.

- all randomized subjects who:
  - receive at least one dose of vaccine, and
  - provide one evaluable serum sample before and one after baseline

(c) Per protocol (PP) population, Immunogenicity.

- all subjects in the ITT population who:
  - receive all doses of vaccine correctly, and
  - have no major protocol violation as defined prior to unblinding

(d) Safety population.

- all subjects who receive at least one dose of vaccine and with some post-baseline safety data.

6.9.1.1 Analysis of Demographic and Baseline Characteristics

Descriptive statistics (mean, standard deviation, median, minimum and maximum) for age, height and weight at enrollment will be calculated overall and by vaccine group.

Distributions of subjects by sex, ethnic origin and previous influenza vaccination will be summarized overall and by vaccine group.

6.9.1.2 Analysis of Immunogenicity Criteria

To evaluate immunogenicity of two 0.5 mL IM injections of FLUAD-like influenza vaccines containing either 7.5 µg or 15 µg of H5N1 influenza antigen, as measured by HI, in compliance with the requirements of the current European Union recommendations (CPMP/BWP/214/96), and by MN and SRH (in a subset of subjects).

Additionally, the immune response as measured by HI assay elicited by two 0.5 mL IM injection of FLUAD-like influenza vaccine containing 7.5 µg of H5N1 influenza antigen will be considered non-inferior to the immune response elicited by two 0.5mL IM injection of FLUAD-like influenza vaccine containing 15 µg of H5N1 influenza antigen, if the lower limit of the 95% CI around the "day 43 GMTs Ratio" ($GMT_{7.5}/GMT_{15}$) is higher than 0.5.

The hypothesis testing will be:

H_0: $\log(GMT_{15}) - \log(GMT_{7.5}) \geq 0.301 \leftrightarrow GMT_{7.5} / GMT_{15} \leq 0.5$ (null hypothesis);

H_1: $\log(GMT_{15}) - \log(GMT_{7.5}) < 0.301 \leftrightarrow GMT_{7.5} / GMT_{15} > 0.5$ (alternative hypothesis);

Where: $GMT_{7.5}$ = GMT for strain H5N1 after two doses of influenza vaccine containing 7.5 µg of H5N1 influenza antigen;
GMT_{15} = GMT for strain H5N1 after two doses of influenza vaccine containing 15 µg of H5N1 influenza antigen.

The measures of immunogenicity will be calculated as:

Geometric Mean Titer or Geometric Mean Area. For each vaccine group, least squares GMTs for HI and MN data (GMAs for SRH data), associated 95% confidence interval and median, minimal, and maximal titer value will be determined for study day 1, for study day 22, for study day 43, for study day 202, for study day 223 and for study day 382, using analysis of variance (ANOVA) with one factor for vaccine group.

Geometric Mean Ratio. For each of the two vaccine groups, the least squares GMRs will be calculated for the HI, MN and SRH results for the following time points of the study: day 22/day 1, day 43/day22, day 43/day 1, day 201/day 43, day 202/day 1, day 223/day 202, day 223/day 1, day 382/day 223, dat 382/day 202, as well as the associated 95% confidence intervals and the median, minimal, and maximal n-fold increase. Statistical methods used to analyze GMRs will be identical to those described above for GMTs (GMAs).

Percentages of Subjects With Seroconversion or Significant Increase in HI Titer and SRH Area. The number and proportion of subjects achieving seroconversion or at least a four-fold increase in HI titers or SRH areas from pre-immunization to 21, 42, 202, 223 and 382 days after first immunization will be tabulated for each vaccine group.

Seroconversion is defined as negative pre-vaccination serum (< 10 for HI, 4 for SRH) / positive post-vaccination titer (≥ 40 for HI, area ≥ 25 mm^2 for SRH).

Significant increase in antibody titer/area is defined as at least a fourfold increase from non-negative pre-vaccination serum (≥ 10 for HI) or a 50% increase in area for SRH.

Percentages of Subjects With at Least a 4-fold Increase in HI, MN Titer or SRH area. The number and proportion of subjects achieving at least a four-fold increase in HI, MN or

SRH from pre-immunization to 21, 42, 202, 223 and 382 days after first immunization will be tabulated for each vaccine group.

Percentages of Subjects Achieving an HI or MN Titer ≥ 40 or an SRH area ≥ 25 mm^2. The number and proportion of subjects achieving HI or MN titers of at least 40 or an SRH area ≥ *25 mm^2* at study day 1, at study day 22, at study day 43, at study day 202, at study day 223 and at study day 382 will be tabulated for each vaccine group.

All tables and listings will be presented by vaccine type and by age group.

All statistical analyses will be performed on the logarithmically (base 10) transformed values.

Titers below the limit of detection for HI and MN assay will be set to half that limit for the purposes of analysis, i.e., 5. The lower detection limit of the SRH test is at an area of 4. All areas below the lower limit of detection will be set to 4 for the immunogenicity analysis. Original values will be presented in all listings.

6.9.1.3 Analysis of Safety Criteria

They include data from the physical examination and observed local and systemic reactions and adverse events.

Local reactions include:

- Ecchymosis, erythema, induration, swelling and pain at injection site.

Systemic reactions include:

- Chills, malaise, myalgia, arthralgia, nausea, headache, sweating, fatigue, and potential indicators of oculo-respiratory syndrome such as: coughing, wheezing, chest tightness, other difficulty breathing, sore throat, facial edema, and red eye. Fever, as a systemic reaction, will be programmatically derived from measured axillary temperatures ≥ 38°C.

Any other indications of reactogenicity, all the adverse events occurring during the first 6 weeks of the study (study day 1 – study day 43) and from study day 202 to study day 223, either judged as related or not to vaccination by the Investigator, will be recorded. All serious adverse events adverse events necessitating a physician's visit and/or resulting in premature subject's withdrawal from the study as well as pregnancies will be collected throughout the study.

Vaccine-related adverse events causing any permanent damage or a transient damage severe enough to affect normal activities or requiring any treatment will be considered as major side effects. Milder vaccine-related adverse events will be considered as minor side effects.

Local and Systemic Reactions. Incidence of local and systemic reactions occurring during 1-7 days after each vaccination will be tabulated.

Erythema, ecchymosis, swelling, and induration will be categorized as none, 1 to ≤ 10 mm, 11 to ≤25 mm, 26 to ≤ 50 mm, and > 50 mm. All other systemic reactions will be categorized as none, mild, moderate, and severe. Each local and systemic reaction will also be categorized as none vs. any.

Other indicators of reactogenicity: Distribution of body temperature, staying at home due to vaccine reaction and the use of analgesic/antipyretic medication occurring during 1-7 days after each vaccination will be tabulated. Axillary temperature will be categorized as < 38°C, 38°C to < 39°C, 39°C to < 40°C, and ≥ 40°C.

Adverse Events: Summaries presenting number of subjects reporting adverse events (AEs) will be prepared. The original verbatim terms used by the investigator to identify adverse events in the CRF will be translated into preferred terms using MedDRA, version 8.1 or higher. The adverse events will then be grouped by preferred terms into frequency tables according to system-organ class (SOC). When an adverse event is reported more than once by the same subject, the maximal severity and worst causality will be used. Adverse events that are considered to be possibly or probably related to study vaccine will be summarized separately. Serious adverse events and adverse events necessitating a physician's visit and/or leading to premature withdrawal from study will be listed. Additionally, adverse events that are unrelated to vaccine will be summarized and data listings of all adverse events will be provided.

6.9.1.4 Interim Analysis Planned

No interim analysis of data from this trial is planned. Should it later become necessary, the analysis will be governed by the procedures specified in the Novartis BCDM standard operating procedure entitled "Interim Analysis in a Clinical Trial".

6.9.1.5 Preliminary Analysis

A preliminary analysis of immunogenicity and safety will be performed when all Visit 3 results are available.

6.9.2 Determination of Sample Size

The sample size calculation is based on the secondary immunogenicity objective of non-inferiority between two 0.5 mL doses of FLUAD-like influenza vaccine containing 7.5 µg of H5N1 antigen vs. two 0.5 mL doses of FLUAD-like influenza vaccine containing 15 µg of H5N1 antigen, as measured by HI test.

A 0.025 one-sided alpha level, a clinically relevant value of 0.5 in terms of the ratio of post-immunization GMTs (day 43, visit 3) between the two vaccine doses (i.e., a difference of 0.301 in terms of log [GMTs] between vaccines) and a power of 80% are chosen.

Assuming a standard deviation of 1.0 (calculated as the upper limit of the 80% CI of the standard deviation reported in a previous Chiron (now Novartis) Vaccines pilot study, V7P37) for both formulations of FLUAD-like influenza vaccine, 230 evaluable subjects per group (460 in total) will be necessary to test the null hypothesis. Considering the drop-outs, a suitable number of subjects should be enrolled in order to achieve at least 460 evaluable subjects.

In total 220 enrolled subjects will be consecutively selected for immunogenicity evaluation by using MN assay and possibly SRH, in order to achieve a subset of at least 200 evaluable subjects (100 per age group, and within them 50 per vaccine type).

6.10 Documentation of Study Findings

A set of CRF collations will be supplied by the sponsor. The CRFs do not require carbon paper. The bottom copy must be retained by the investigator, and all other copies will be returned as directed by the sponsor. Instructions on how to complete these forms will be given to the investigator.

All study findings must be entered by the investigator on these CRFs. If the investigator authorizes other persons to make entries on the CRF, the names, positions, signatures and initials of these persons must be supplied to the sponsor.

All entries in the CRFs must be made *legibly* in black ball-point pen (*not pencil, felt tip or fountain pen*).

The following data may be reported directly on the CRFs and are considered to be source data: demographics data, medical history, vaccination time, adverse events, concomitant medication, study termination and comments.

For each enrolled subject a CRF collation must be signed and dated by the investigator named in the study protocol. CRFs must be completed immediately after the final examination. Arrangements will be made by the monitor to collect the CRFs on completion. No CRFs are to be mailed to the sponsor without specific authorization.

A reasonable explanation must be given by the investigator for all missing data.

If corrections are made to entries in the CRF by the investigator or designates, the words or figures must be crossed through, leaving the initial entry legible. The correction must then be dated and initialed. Incorrect entries must not be covered with correcting fluid, obliterated, or made illegible in any way. If further corrections are made after review and signature by the investigator, he/she must be made aware of the changes and document this awareness.

As part of the conduct of the trial, Novartis may have questions about the case report form data after the CRFs are collected from the site. These questions will be documented using

Data Clarification Forms (DCFs). Novartis will answer the question if the answer is self-evident on “internal” DCFs, but the investigator will need to review these changes. If the query is not self-evident, Novartis will ask the investigator to provide the answer on an “external” DCF and sign it. Novartis will send the investigator a copy of each completed internal DCF. The investigator will review them and if he/she disagrees with the resolution, he/she will notify the site monitor, who will then generate a new DCF documenting the investigator’s correction. The investigator will be requested to file each of the DCFs (both levels) for the trial.

Definitions of the DCF types are as follows:

Internal DCF: A query for which the resolution is self-evident. It can be answered without changing the meaning of the data (such as moving data from one box to another) or using logical numeric flow (for example, if an adverse event number is missing, the next number in sequence can be assigned). Internal corrections are defined in either the assumptions for internal queries guideline or in study-specific assumptions. The Clinical Data Coordinator or the Clinical Research Associate can answer an internal query. Key safety (e.g. adverse event relationship) or primary endpoint data queries are not to be handled as an internal DCF.

External DCF: A query that requires information from the investigator. Key safety (e.g. adverse event relationship) or primary endpoint data queries must be handled as an external DCF.

6.11 Record Retention

Investigators must retain all study records required by Novartis and by the applicable regulations in a secure and safe facility. The investigator must consult a Novartis representative before disposal of any study records, and must notify the sponsor of any change in the location, disposition, or custody of the study files. “Essential documents” are defined as documents that individually and collectively permit evaluation of the conduct of a trial and the quality of the data produced. Essential documents must be retained until at least 2 years after the last approval of a marketing application in an ICH region and until there are no pending or contemplated marketing applications in an ICH region or at least 2 years have elapsed since the formal discontinuation of clinical development of the investigational product. The Committee for Human Medicinal Products (CHMP) requires retention for the maximum period of time permitted by the institution, but not less than 15 years. It is the responsibility of the sponsor to inform the investigator/institution as to when these documents no longer need to be retained.

6.12 Use of Information and Publication

It is understood by the investigator that the information generated in this study will be used by the sponsor in connection with the development of the product and therefore may be disclosed to government agencies in various countries. To allow for the use of

information derived from the study, it is understood that the investigator is obliged to provide the sponsor with complete test results, all study data, and access to all study records.

The sponsor recognizes the importance of communicating medical study data and therefore encourages their publication in reputable scientific journals and at seminars or conferences.

Any results of medical investigations with the sponsor products and/or publication/lecture/ manuscripts based thereon, shall be exchanged and discussed by the investigator and the sponsor Clinical Research representative(s) 60 days prior to submission for publication or presentation. Due regard shall be given to the sponsor's legitimate interests, e.g., manuscript authorship, obtaining optimal patient protection, coordinating and maintaining the proprietary nature of submissions to health authorities, coordinating with other ongoing studies in the same field, and protecting confidential data and information.

In cases of publications or presentations of material arising from multicenter clinical investigations, the sponsor is to serve as coordinator and referee. Individual investigators who are part of a multicenter investigation may not publish or present data that are considered common to a multicenter investigation without the consent and the prior review of the sponsor. In case of disagreement amongst the authors, the sponsor will be the final arbiter. The sponsor comments shall be given without undue delay. If they are not accepted, the senior author of the manuscript and the sponsor's representatives shall promptly meet to discuss further and endeavor to agree mutually on the final wording and/or disposition of the publication. The above procedure also applies to information on prematurely discontinued and other not completed studies.

Results from investigations shall not be made available to any third party by the investigating team outside the publication procedure as outlined previously. The sponsor will not quote from publications by investigators in its scientific information and/or promotional material without full acknowledgment of the source (i.e., author and reference).

All information concerning the study drug supplied by the sponsor in connection with this clinical study, and not previously published, is considered confidential and proprietary information. This information includes the Investigator's Brochure, clinical protocol, workbooks if applicable, CRFs, assay methods, the sponsor's technical methodology, and basic scientific data. This confidential information shall remain the sole property of the sponsor, shall not be disclosed to others without prior written consent from the sponsor and shall not be used except in the performance of this clinical study.

The information developed during the conduct of this clinical study is also considered confidential and will be used by the sponsor in connection with the development of the study drug. This information may be disclosed as deemed necessary by the sponsor.

The investigator is obliged to provide the sponsor with complete test results and all data derived from this clinical study. Only the sponsor may make information obtained during this clinical study available to the physicians and to the regulatory agencies, except as required by regulation.

7. REFERENCE LIST

1. TREANOR J. Influenza Vaccine – Outmaneuvering Antigenic Shift and Drift. NEJM 350;3. January 15, 2004: 218-220

2. NGUYEN-VAN-TAM JS, HAMPSON AW. The epidemiology and clinical impact of pandemic influenza. Vaccine 21(2003): 1762-1768

3. SIMONSEN L, SCHONBERGER LB, STROUP DF, ARDEN NH, COX NJ. Impact of influenza on mortality in the USA. In: BROWN LE, HAMPSON AW, WEBSTER RG, eds. Options for the control of influenza III: proceedings of the 3rd International Conference on Options for the Control of Influenza, Cairns, Australia, 4-9 May, 1996. Amsterdam, Holland: Elsevier Science, 1996: 26-33

4. Weekly Epidemiological Record (2002) 77:394-404

5. STOHR K. The Global Agenda on Influenza Surveillance and Control. Vaccine 21(2003): 1744-1748

6. Prevention and Control of Influenza Pandemics and Annual Epidemics. Resolution of the 56th World Health Assembly, 28 May 2003

7. FEDSON DS. Pandemic Influenza and the Global Vaccine Supply. Vaccines. CID 2003:36 (15 June); 1552-1561

8. WEBBY RJ, WEBSTER RG. Are we ready for pandemic influenza? Science, Vol 32. 28 November 2003: 1519-1522

9. Development of a Clinical Trial Plan for Pandemic Influenza Vaccines. Department of Health and Human Services. National Institutes of Health/NIAID Workshop, Bethesda, USA, September 22-23, 2003

10. TRAN TH, NGUYEN TL, NGUYEN TD, LUONG TS, PHAM PM, VAN NGUYEN VC ET AL. Avian influenza A(H5N1) in 10 patients in Vietnam. NEJM 2004;350:1179-88

11. WHO informal meeting on influenza pandemic vaccine development. Geneva 13 February 2004

12. ENSERINK M. Pandemic vaccines appears to protect only at high doses. Science. 2005; 309: 996.

13. NICHOLSON KG, COLEGATE AE, PODDA A, et al. Safety and antigenicity of non-adjuvanted and MF59-adjuvanted influenza A/Duck/Singapore/97 (H5N3) vaccine: a randomised trial of two potential vaccines against H5N1 influenza. The Lancet. 2001;357:1937-1943

14. STEPHENSON I, NICHOLSON KG, COLEGATE A, et al. Boosting immunity to influenza H5N1 with MF59-adjuvanted H5N3 A/Duck/Singapore/97 vaccine in a primed human population. Vaccine. 2003;21:1687-1693.

8. INVESTIGATOR AGREEMENT

I have read the preceding protocol

"A Phase II, Randomized, Controlled, Observer-blind, Multi-Center Study to Evaluate Safety and Immunogenicity of Two Doses, Administered Three Weeks Apart, and a Six Months Booster Dose of Two FLUAD-like (Surface Antigen Adjuvanted with MF59C.1) Influenza Vaccines Containing 7.5 µg or 15 µg of H5N1 Influenza Antigen, in Non-elderly Adult and Elderly Subjects"

and received the following:

- Investigator's Brochure with details of pharmacological and toxicological findings with the investigational product, dated 22 DEC 05.
- "ICH Harmonized Tripartite Guideline for Good Clinical Practice".

I have been adequately informed about the development of the investigational product to date. I have read this study protocol and agree that it contains all the information required to conduct the study. I agree to conduct the study as set out in this protocol.

I will ensure that all persons assisting with the trial are adequately informed about the protocol, any amendments to the protocol, the study treatments, and their trial-related duties and functions. I will maintain a list of sub-investigators and other appropriate qualified persons to whom I have delegated significant trial-related duties.

I will provide copies of the study protocol and all drug information relating to preclinical and prior clinical experience furnished to me by the sponsor, to all physicians responsible to me who participate in this study. I will discuss this material with them to assure that they are fully informed regarding the vaccine and the conduct of the study.

I will not enroll the first subject in the study until I have received approval from the appropriate EC, where applicable, and until all legal requirements in my country have been fulfilled.

I will provide a curriculum vitae before the study starts. I agree that these data may be submitted, if necessary, to the relevant authorities. I further agree that my name and contact details and those of my staff will be entered into an electronic study management system maintained by the sponsor for internal use only.

Signing this protocol I agree

- to conduct this clinical study in compliance with ICH GCP, with the applicable regulatory requirement(s), and with the study protocol agreed to by the sponsor and given approval/favorable opinion by the EC

- to comply with procedures for data recording/reporting
- to permit monitoring, auditing, inspection, and EC review and

to retain the study related essential documents according to legal requirements and as agreed with the sponsor.

Coordinating Investigator

....
(print name)

....
(place, date) (signature)

Principal Investigator

....
(print name)

....
(place, date) (signature)

Principal Investigator

....
(print name)

....
(place, date) (signature)

Principal Investigator

....
(print name)

....
(place, date) (signature)

9. SPONSOR AGREEMENT

This study protocol was subject to critical review and has been approved by the appropriate Study Document Review Committee. The information it contains is consistent with:
- Investigator's Brochure with details of pharmacological and toxicological findings with the investigational product, dated: 22 DEC 05.
- the moral, ethical and scientific principles governing clinical research as set out in the Declaration of Helsinki and good clinical practice guidelines.

The investigator will be supplied with details of any significant or new findings, including adverse events.

NOVARTIS VACCINES

Global Head of Clinical Research & Medical Affairs

....
(place, date) *Audino Podda, MD*

Medical Monitor

....
(place, date) *Angelika Banzhoff, MD*

Study Monitor

....
(place, date) *Massimo Bianchini, PhD*

Head of Clinical Biostatistics

....
(place, date) *Pantaleo Nacci, DStat*

Study Biostatistician

....
(place, date) *Sandrine Tilman, MSc*

APPENDIX A
LIST OF CENTRAL LABORATORIES

The HI and MN tests described in the protocol will be performed at the laboratory of:
- Clinical Serology, Novartis Vaccines, Gebäude Z26, Emil-von-Behring Str. 76, 35041 Marburg, Germany.

The laboratory for the SRH test is:
- Laboratorio di Epidemiologia Molecolare, Dipartimento di Fisiopatologia, Medicina Sperimentale e Sanità Pubblica, Università di Siena, Siena, Italy.

APPENDIX B
SHIPPING AND HANDLING OF LABORATORY SPECIMENS

1. Serum must be centrifuged, separated and frozen as soon as possible after collection. Make sure that coagulation is complete before separating the serum from blood components.

2. Please use the cryotubes provided. Be aware that the maximum-fill volume for these tubes is 90 % of rated capacity, i.e., 3.6 mL, to allow for expansion of the frozen serum. The tubes are marked with a maximum-fill line. Other sizes of cryotubes are not acceptable; if you have an inadequate supply, please contact your Monitor and additional tubes will be provided.

3. Close the tubes before freezing, and check to make sure that the caps are completely tight before packing for shipment.

4. Barcode labels are to be placed lengthwise down the tubes (not wrapped around). Barcodes are machine-readable only when their entire length runs along a flat surface. Please do not write on, make markings through, or otherwise deface the barcode. Make sure that labels are securely attached so that they do not pull away from the tubes after freezing. It is not necessary to fasten the label to the tube with adhesive tape.

5. Before the start of the trial, prepare separate cryoboxes for original and duplicate samples (“Original “ and “Duplicate” for all subjects and “Original”, “Original 2” and “Duplicate” labeled cryotubes for the first 28 subjects of each age group at each site). After serum has been collected, centrifuged and separated, place labeled tubes in the respective labeled cryoboxes “Original“ and “Duplicate”. Keep the tubes in numerical order by subject number.

6. Freeze tubes at ≤ -18 °C (to -80 °C), in an upright position so that serum does not come into contact with the cap. Tubes may be stored in any convenient position once they are frozen. Do not "quick-freeze" tubes by immersing them in either dry ice or liquid nitrogen; this may crack the tubes.

7. Pack cryotubes for shipment according to the instructions provided. If you have questions, please contact your Monitor.

8. Pack the shipping logs in an envelope inside the cardboard shipping boxes, on top of the Styrofoam insulated shipper. Keep a copy of each log at the site for future reference and send the “Original” and the “Original 2” logs together with the “Original” and the “Original 2”samples to:

Dr. Anne Katrin Hilbert
Novartis Vaccines
Klinische Serologie

Gebäude Z26
Emil-von-Behring Str. 76
35041 Marburg
Germany
Phone number: +49 (0)6421 393439

9. The “duplicate” samples should be sent still frozen after confirming that the “original” and “original 2” shipments arrived safely.

APPENDIX C
SEROLOGY PROCEDURES

The laboratory procedures for this study will be based on the European “General requirements for the competence of testing and calibration laboratories” (ISO/IEC 17025).

Retention of Study Records

The study protocol, raw data and other documents generated during the course of the study will be retained in: Clinical Serology, Novartis Vaccines, Marburg, Germany and Laboratorio di Epidemiologia Molecolare, Dipartimento di Fisiopatologia, Medicina Sperimentale e Sanità Pubblica, Università di Siena, Siena, Italy for 15 years.

Quality Assurance Reviews

Quality Assurance of operations that are routinely performed may be carried out by means of process-based inspections. Technical review on all recorded stages of the assay data will be performed.

Objectives

To test human sera for the presence of neutralizing antibodies to
H5N1 and, possibly, other strains.

Sample Storage

After the performance of the HI, and MN **and possibly SRH** tests described in this protocol, all serum samples should be logged and stored at a temperature of minus 18°C or below in the respective laboratories.

Test Methods and Procedures

HI-Flu H5N1

SRH-Flu H5N1

Reference: CPMP/BWP/214/96

MN-Flu H5N1

Equipment

Equipment as listed in the relevant Clinical Serology, Novartis Vaccines, Marburg and **Laboratorio di Epidemiologia Molecolare, Dipartimento di Fisiopatologia, Medicina Sperimentale e Sanità Pubblica, Università di Siena** SOP and/or associated documents.

Procedures

All procedures will be carried out according to the relevant Clinical Serology, Novartis Vaccines, Marburg **and Laboratorio di Epidemiologia Molecolare, Dipartimento di Fisiopatologia, Medicina Sperimentale e Sanità Pubblica, Università di Siena**, laboratory SOP(s) and/or associated document(s).
